# UHPLC/MS‐MS Profiling, Antioxidant and Antimicrobial Properties of *Atriplex halimus* L. and *Marrubium deserti* De Noé Extracts: In Vitro and In Silico

**DOI:** 10.1002/cbdv.71807

**Published:** 2026-10-09

**Authors:** Nacira Siad, Chahrazed Hamia, Amar Djeridane, Mohamed Benalia, Reguia Mahfoudi, Hicham Bouakkaz, Mustafa Abdullah Yilmaz, Oguz Cakir, Abbas Tarhan, Mohamed Yousfi

**Affiliations:** ^1^ Laboratoire Des Sciences Fondamentales Amar Telidji University Laghouat Algeria; ^2^ Laboratory of Applied Sciences and Didactic Higher Normal School Laghouat Algeria; ^3^ Science and Technology Research and Application Center Dicle University Diyarbakir Türkiye; ^4^ Department of Analytical Chemistry Faculty of Pharmacy Dicle University Diyarbakir Türkiye; ^5^ Department of Nutrition and Dietetics Faculty of Health Sciences Dicle University Diyarbakir Türkiye

**Keywords:** antimicrobial capacity, antioxidant activity, *Atriplex halimus* L., *Marrubium deserti* De Noé, molecular docking, phenolic compounds

## Abstract

This study aims to evaluate the chemical composition and both in vitro and in silico antioxidant and antimicrobial activities of *Atriplex halimus* L. and *Marrubium deserti* De Noé, collected from Algeria. UHPLC‑MS/MS analysis revealed that the major compounds in the tested extracts are protocatechuic aldehyde, caffeic acid, cynaroside, and cosmosiin. The data demonstrated that the dichloromethane fraction was richer in polyphenols and flavonoids than the aqueous fraction. The extracts exhibited greater antioxidant capacity against ABTS compared to DPPH and FRAP assays. Antimicrobial activity was assessed by determining MIC_50%_, MIC_90%_, and MBC using a technique that correlates the colorimetric method with absorbance and concentration. The results showed that the extracts strongly inhibited all tested microorganisms, with MIC_50%_, MIC_90%_, and MBC values ranging from 0.0029 to 4.485 mg/mL, 0.0054 to 8.693 mg/mL, and 0.006 to 9.659 mg/mL, respectively. Furthermore, the extracts demonstrated notable efficacy comparable to antibiotics, especially against *Candida albicans* and *Pseudomonas aeruginosa*. These in vitro findings were supported by computational analyses. These methods provide predictive evidence regarding the plausibility of the observed biological effects of the extracts. The results of this research suggest that these Algerian plants exhibit significant therapeutic potential and should be considered for nutritional and medicinal applications.

## Graphical abstract

This study investigates the phytochemical profile and biological potential of *Atriplex halimus* L. and *Marrubium deserti* De Noé extracts. Polyphenolic constituents are identified using UHPLC‐MS/MS analysis, complemented by TPC and TFC evaluations. Antioxidant activities are determined via DPPH, ABTS, and FRAP assays, while antimicrobial efficacy is evaluated against *Staphylococcus aureus*, *Pseudomonas aeruginosa*, *Escherichia coli*, and *Candida albicans* through MIC_50%_ and MIC_90%_, MBC, and MFC determinations. Furthermore, computational analysis and statistical modeling are applied to assess bioactivity mechanisms and correlations.

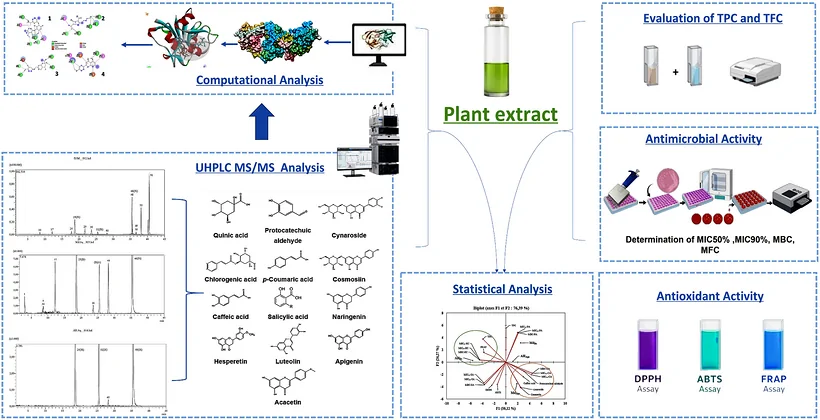

## 1 Introduction

Natural antioxidants and antimicrobial sources are essential for health enhancement and disease inhibition, especially in the face of antibiotic resistance and oxidative stress [1]. Bioactive compounds found in plants, herbs, and other natural materials, such as polyphenols, flavonoids, alkaloids, and essential oils, exhibit strong antioxidant activity and antimicrobial properties [2]. These compounds offer safer, more sustainable alternatives to synthetic drugs and are deeply rooted in traditional medicine [3]. Modern research validates their therapeutic potential in treating infections, inflammation, and chronic diseases. Their low toxicity and biodegradability make them valuable assets in healthcare and pharmaceutical development [4].

Algerian medicinal plants are widely acknowledged to contain biologically active compounds that have diverse pharmacological properties, particularly antioxidant and antimicrobial effects [5]. Among these rich plants, *Atriplex halimus L*. and *Marrubium deserti* De Noé hold significant importance in Algerian traditional medicine for treating various diseases.

*Atriplex halimus* L. (Amaranthaceae), commonly known as Elgettaf, is a resilient shrub widely found in Algeria's dry and semi‐arid regions. It is recognized for its ecological and medicinal significance [6]. Traditionally used in Algerian traditional medicine, it has been employed to treat a variety of ailments, counting diabetes, urinary tract infections, digestive disorders, and rheumatism [7]. The chemical composition of this plant is defined by its standing as a salt plant, characterized by essential oils rich in biologically active terpenes such as phytol and viridiflorol, which provide the plant with characteristic antioxidant and antimicrobial properties [8]. Its phytochemical matrix is also enhanced by a variety of flavonoids, including quercetin, kempferol, and isorhamnitine derivatives, along with phenolic acids such as caffeic acid and ferulic acid that contribute to its anti‐diabetic and anti‐inflammatory activity [9]. In addition, the plant contains significant levels of saponins and specialized osmoregulators such as glycine betaine, which are essential for its survival in soil with high salinity and contribute to its traditional use in treating urinary tract and digestive system disorders. Additionally, *Atriplex halimus* L. contributes to environmental sustainability by stabilizing soils and serving as livestock fodder, making it valuable both medicinally and agriculturally [10].

*Marrubium deserti* De Noé member of (Lamiaceae) is commonly known as Eljaida, grows in the desert of Algeria, is a medicinal plant with a profound phytochemical profile and therapeutic uses [11]. This plant possesses powerful antimicrobial and antifungal properties due to a complex array of natural chemical constituents. Its effectiveness is rooted in the presence of several organic compounds, including unique biologically active molecules like marrubiin, desertine, and various cyllenins and marrulibacetals. The plant defense system is reinforced by a high concentration of phenolic compounds, specifically flavones such as apigenin and its glycosides, flavonols like quercetin and rutin, and various phenolic acids including gallic and trans‐cinnamic acid, equipping the plant with a strong natural resistance against microbial and fungal threats [12, 13]. It has also been shown to disturb microbes as well as yeasts, to a lesser degree, with the predominant inhibition of the germination of microorganisms and sporulation [14]. In Algeria *Marrubium deserti* De Noé is used to remedy fever, colds, colic, cough, jaundice, high blood pressure, and diabetes [15]. The plant is also used in the treatment of allergic reactions and scorpion stings. It has high antioxidant activity and adds to the traditional medicine due to its anti‐inflammatory and analgesic activities [16].

On the other hand, molecular docking has emerged as a crucial technique in drug development. This approach utilizes specialized computational tools to predict and analyze molecular interactions. By simulating binding behaviors, it enables researchers to estimate interaction strength, optimize therapeutic efficacy, and reduce overall development costs [17].

To date, traditional knowledge of *Atriplex halimus* L. and *Marrubium deserti De Noé with* modern molecular insights are not yet linked by a comprehensive scientific validation. In this context, the present study provides a multi‐level characterization of these two species by studying their chemical constituents using UHPLC/MS‐MS analysis of four extracts and their antioxidant and antimicrobial properties through both in vitro and in silico molecular studies, in order to provide further evidence for their traditional use in Algerian popular medicine.

## 2 Materials and Methods

### 2.1 Biological Materials

Sterile wire loop, sterile normal saline solution, petri plates, sterile swab, gentamicin (80 mg/2 mL), imipenem (500 mg), antifungal fluconazol (200 mg/100 mL), sterile distilled water, Sabouraud agar, nutrient agar, Mueller–Hinton agar, Iodonitrotetrazolium chloride (INT), test tubes, 96‐well microplates, micropipette, Pasteur pipette.

### 2.2 Chemical Materials

The experiments used analytical grade chemical products and solvents from Sigma‐Aldrich (USA and France), and Honeywell (Germany). methanol(CH_3_OH), ethanol(C_2_H_6_O), dimethyl sulfoxide (DMSO), hydrochloric acid solution (37%), ammonium hydroxide (H_5_NO), dichloromethane DMC (CH_2_Cl_2_), acetic acid(CH_3_COOH), sodium carbonate (Na_2_CO_3_), gallic acid(C_6_H_2_(OH)_3_COOH), Folin–Ciocalteu reagent, sodium sulfate (Na2SO4), diammonium 2,2′‐azino‐bis(3‐ethylbenzothiazoline‐6‐sulfonate)(ABTS), potassium persulfate K_2_S_2_O_8_, di sodium hydrogen phosphate dodehydrate (Na_2_HPO_4_·12H_2_O), sodium phosphate monobasic dihydrate (NaH_2_PO_4_·2H_2_O), aluminum trichloride (AlCl_3_).sodium chloride (NaCl), 2,2‐Diphenyl‐1‐(2,4,6‐trinitrophenyl)hydrazyl(DPPH), 2,4,6‐Tris(2‐pyridyl)‐s‐triazine (TPTZ), ferric chloride hexahydrate(FeCl_3_·6H_2_O), acetic acid sodium salt trihydrate(C_2_H_3_NaO_2_·3H_2_O), ascorbic acid.

### 2.3 Plant Material Collection

Aerial parts of *Atriplex halimus L*. (*A. halimus*) and *Marrubium deserti De Noé (M. deserti)* were collected from the Sidi Makhlouf community (34.133333°N, 3.016667°E), located 40 kilometers from Laghouat, Algeria. Professor Amar Djeridane authenticated the plant specimens. The verified samples, designated *AH‐SM‐22* and *MD‐SM‐22*, were subsequently deposited in the research herbarium of the Fundamental Science Laboratory at the University of Laghouat. After collection, the samples were air‐dried in the dark at ambient temperature. Fresh plant material was placed in a dry, well‐ventilated, and light‐protected area for one month to ensure complete drying.

### 2.4 Preparation of Plant Extracts

The collected plant samples underwent grinding and were then sieved through a 200 µm mesh. Subsequently, five grams (5 g) of the sieved material were subjected to extraction with 100 mL of methanol (Me) in an ultrasonic bath for 30 min at 37°C. The resulting extract was filtered using 8 µm filter paper, and a rotary evaporator was used to concentrate the filtrates to dryness under vacuum at 45°C. The residual material was reconstituted in 30 mL of a 1 M acidic aqueous solution, then extracted using an equal volume of dichloromethane through liquid–liquid separation. The organic fraction was dried over sodium sulfate, filtered, and concentrated to dryness using a rotary evaporator. To process the remaining aqueous fraction, 7 mL of ammonium hydroxide was added gradually until the pH reached 10. This alkaline solution was then extracted with 37 mL of dichloromethane. The resulting organic fraction underwent the same drying and filtration steps, followed by evaporation to remove the solvent. The final aqueous portion was also evaporated to dryness using the rotary. Finally, for subsequent usage, the residues from the aqueous and organic phases were redissolved in appropriate volumes of DMSO, to achieve final concentrations equal to 10 mg/mL.

### 2.5 Estimation of Total Phenolic Contents

The total phenolic content (TPC) was estimated by a modified spectrophotometric method, as previously described by Singleton & Rossi [18], which employs the Folin–Ciocalteu reagent. 100 µL of the sample solutions were combined with 500 µL of Folin–Ciocalteu reagent 10% diluted. Following a 2 min incubation period, the mixture was mixed with aqueous sodium carbonate solution (2%). After another 30 min of incubation, the absorbance of the solution was read at 760 nm, with a calibration curve prepared using varying concentrations of gallic acid as a reference. The findings were presented as (mg GAE g^−1^ extract).

### 2.6 Estimation of Total Flavonoid Contents

The spectrophotometric method using aluminum chloride (AlCl_3_), as defined by Djeridane et al. [19], was utilized to estimate total flavonoid content (TFC). 1 mL of a 2% aluminum chloride solution was combined with 1 mL of the diluted sample. After 20 min of incubation, the absorbance at 430 nm was measured. Then, quercetin served as the calibration standard, and the flavonoid content was expressed as (mg QE g^−1^ extract).

### 2.7 HPLC MS/MS Analyses

A Shimadzu–Nexera model ultrahigh‐performance liquid chromatograph (UHPLC) coupled with a tandem mass spectrometer was used to quantitative of phytochemicals in the samples [20, 21]. The reversed‐phase UHPLC was equipped with an autosampler (SIL‐30AC model), a column oven (CTO‐10ASvp model), binary pumps (LC‐30AD model), and a degasser (DGU‐ 20A3R model). The chromatographic conditions were optimized in order to achieve optimum separation for 53 phytochemicals and overcome the suppression effects. Different columns such as Agilent Poroshell 120 EC‐C18 model (150 mm × 2.1 mm, 2.7 µm) and RP‐C18 Inertsil ODS‐4 (100 mm × 2,1 mm, 2 µm), different mobile phases (B) such as acetonitrile and methanol, different mobile phase additives such as ammonium formate, formic acid, ammonium acetate, and acetic acid, different column temperatures such as 25°C, 30°C, 35°C and 40°C were tried and applied until the optimum conditions were achieved. Consequently, the chromatographic separation was performed on a reversed‐phase Agilent Poroshell 120 EC‐C18 model (150 mm × 2.1 mm, 2.7 µm) analytical column. The column temperature was set to 40°C. The elution gradient was composed of eluent A (water + 5 mM ammonium formate + 0.1% formic acid) and eluent B (methanol + 5 mM ammonium formate + 0.1% formic acid). The following gradient elution profile was used: 20%–100% B (0–25 min), 100% B (25–35 min), 20% B (35–45 min). Furthermore, the solvent flow rate and injection volume were set to 0.5 mL/min and 5 µL, respectively.

The mass spectrometric detection was carried out using a Shimadzu LCMS‐8040 model tandem mass spectrometer equipped with an electrospray ionization (ESI) source operating in both negative and positive ionization modes. LC‐ESI‐MS/MS data were acquired and processed by LabSolutions software (Shimadzu). The MRM (multiple reaction monitoring) mode was used for the quantification of the phytochemicals. The MRM method was optimized to selectively detect and quantify phytochemical compounds based on the screening of specified precursor phytochemical‐to‐fragment ion transitions. The collision energies (CE) were optimized in order to generate optimal phytochemical fragmentation and maximal transmission of the desired product ions. The MS operating conditions were applied as: drying gas (N_2_) flow, 15 L/min; nebulizing gas (N_2_) flow, 3 L/min; DL temperature, 250°C; heat block temperature, 400°C, and interface temperature, 350°C [22, 23].

### 2.8 Antioxidant Activity

The antioxidant activity of the extracts was estimated by DPPH, ABTS, and FRAP assays. Results are reported as the 50% efficacy concentration (EC_50_) in mg/L.

### 2.9 Free‐Radical Scavenging Activity (DPPH assay)

The scavenging capacity of the extracts against free radicals was determined using a slightly modified DPPH assay, based on the method described by Valko et al. [24]. The experimental setup involved preparing a mixture containing 1 mL of a 125 µM ethanolic solution of DPPH and 1 mL of either the sample extract or the reference standard. After allowing the reaction to proceed for 30 min, the decrease in absorbance was measured spectrophotometrically at 517 nm.

### 2.10 Ferric Reducing Antioxidant Power (FRAP assay)

The ferric reducing antioxidant power (FRAP) assay was performed with slight adjustments to the protocol originally described by Benzie and Strain [25]. The evaluation involved mixing 1 mL of the test extract (or a standard solution of ascorbic acid at different concentrations) with 1 mL of the prepared FRAP reagent. This resulting mixture was incubated in the dark for a period of 7 min. Finally, the amount of the colored product formed, specifically the ferrous tripyridyl‐triazine (TPTZ‐Fe^2^^+^) complex, was quantified by measuring its absorbance at a wavelength of 593 nm

### 2.11 ABTS Cation Radical Scavenging Activity (ABTS•+ assay)

The ability to neutralize the ABTS radical was determined using a slightly modified version of the technique established by Re et al. [26]. The procedure involved combining 100 µL of the test extract (or a standard reference compound) with 900 µL of a freshly prepared, diluted ABTS solution. Following this mixture, it was allowed to react for 15 min at an elevated temperature of 37°C. After the incubation period, the residual absorbance was quantified at a wavelength of 734 nm.

### 2.12 Antimicrobial Activity

The antimicrobial capacity of the extracts was assessed by an in vitro study against four reference microbes sourced from the Pasteur Institute in Algeria: The Gram‐positive bacterium *Staphylococcus aureus* (*S. aureus*) (ATCC 25923), two Gram‐negative bacteria, *Pseudomonas aeruginosa* (*P. aeruginosa*) (ATCC 27853) and *Escherichia coli* (*E. coli*) (ATCC 259221), and the yeast *Candida albicans* (*C. albicans*) (ATCC 10231). For comparative purposes, gentamicin and imipenem were employed as standard antibiotic controls for the three bacterial species, while fluconazole served as the standard antifungal control for *C. albicans*.

The antimicrobial efficacy of the samples was ascertained using a modified broth microdilution method, following the technique outlined by Veiga et al. [27]. The procedure involved dispensing 100 µL of Mueller–Hinton broth for bacterial tests (or Sabouraud broth for the yeast) into each well of the microplate. Next, 100 µL of the extract or antibiotic solution was added to the first well of each column. A serial dilution was then performed by transferring 100 µL sequentially to the adjacent wells, resulting in final dilution factors ranging from 1:2 to 1:256. The test wells received 100 µL of microbial inoculum adjusted to a 0.5 McFarland turbidity standard, while the control wells (blanks) were filled with 100 µL of 0.9% saline solution. The plates were then incubated for 24 h at 37 C°. Following incubation, 40 µL of an INT solution (0.2 g/L) was added to each well, and after a further 30 min incubation period, the resulting absorbance was measured at 540 nm using a microplate reader (Mindray MR‐96A). Then, to calculate the inhibitory concentrations IC_50_, IC_90_ and the minimum bactericidal or fungicidal concentration MBC, MFC. The values obtained were applied to draw a straight‐line equation f(C(mg/mL) = % (inhibition percentage).

### 2.13 Computational Analysis

#### 2.13.1 Biological Activity Prediction (PASS)

PASS, or prediction of activity spectra for substances, is a computational tool that predicts the biological activity of chemical compounds based on their molecular structure. It analyzes known biologically active substances to estimate the likelihood that a new compound will exhibit various pharmacological effects, as well as potential toxic or adverse effects [28]. Using the PASS web server [29] to identify key characteristics of 13 compounds from *A. halimus* and *M. deserti*, extracts. The analysis utilized structural molecular formulae (SMILES) from the PubChem database [30], resulting in a ranked list of potential biological activities, with outcomes labeled as Pa (active) and Pi (inactive).

#### 2.13.2 ADMET Evaluation

ADMET studies, which stand for absorption, distribution, metabolism, elimination, and toxicity, are a crucial part of drug discovery and development. It is estimated that nearly half of medication candidates fail due to insufficient efficacy, and up to 40% fail due to toxicity‐related issues [31]. We evaluated the ADMET properties of the main constituents of *A. halimus* L. and *M. deserti*, extract using pkCSM [32], SwissADME [33], and ADMETlab 3.0 [34].

#### 2.13.3 Molecular Docking

The molecular docking study was used to assess the antioxidant and antimicrobial properties of the compounds derived from *A. halimus* and *M. deserti* extracts against: MecR1 of *S. aureus* (PDB: 6o9s), LpxC from *P. aeruginosa* (PDB: 6dui), GyrB24 of *E. coli* (PDB: 7p2m), Fur1p from *C. albicans* (PDB: 7rh8). Also, against some human oxidoreductases, AKR1B1 (PDB: 8fh8), FOL (PDB: 5sss) and a target for diseases involving oxidative stress, Nrf2 (PDB: 5fzn). However, the three‐dimensional (3D) structures of the phytocompounds were retrieved from the PubChem database and converted from 3D sdf to pdb format utilizing PyMol.

To investigate the interactions between target proteins and their ligands, we obtained their crystallographic structures from the Protein Data Bank [35, 36]. Using the appropriate PDB IDs and visualized them in Discovery Studio v2025 to evaluate protein stability. The preparation of the proteins followed a systematic three‐step procedure that included adding polar hydrogen bonds and Kollman charges, as well as removing water molecules, to enhance docking accuracy. The in silico molecular docking was executed using AutoDock Vina v1.5.6. The protein and the ligand data were converted into PDBQT files through MGL Tools, and the Auto Grid tool was employed to generate grid maps that illustrate the energy of interaction between the target macromolecules and the ligands. The docking protocol was validated by redocking the crystallized ligand into its binding site, and the method's reliability was confirmed through the attainment of a root mean square deviation (RMSD) value below 2 Å. The results of the docking studies were subsequently imported into Discovery Studio Visualizer Version 2025 for comprehensive analysis. All parameters of the proteins are shown in Table 1.

**TABLE 1:** Protein's parameters used in the docking study.

Target	PDB ID	Resolution (A°)	Co‐crystal ligand	Center (X, Y, Z)	Grid size (X, Y, Z)
**MecR1**	**6O9S**	1.59	NXL	(67, 77, 154)	(15, 16, 13)
**LpxC**	**6DUI**	1.55	J1M	(−5, 11, 11)	(14, 17, 14)
**GyrB24**	**7P2M**	1.16	N1N	(−18, −14, 7)	(14, 17, 13)
**Fur1p**	**7RH8**	1.69	UTP	(−27, −58, −8)	(10, 16, 12)
**AKR1B1**	**8FH8**	1.60	NAP	(14, 9, 4)	(25, 25, 26)
**FOL**	**5SSS**	1.14	NAP	(22, 31, 34)	(27, 27, 27)
**Nrf2**	**5FZN**	1.97	FB2	(14, 67, 31)	(25, 25, 25)

#### 2.13.4 Statistical Analysis

Excel 2018 was used to analyze the test data, which were presented as mean ± standard deviation (SD), calculated from triplicate independents experiments. Principal component analysis (PCA) and a 95% confidence interval for the Pearson correlation coefficient were used in the descriptive analysis, which was conducted using XLSTAT 2018.5.03. Tukey's test and one‐way ANOVA were employed to evaluate group means.

## 3 Results and Discussion

### 3.1 Total Phenolic and Flavonoid Contents of Extracts and Their Phenolic Profile

The phenolic and flavonoid contents vary significantly (*p* < 0.001) based on the plants (*A. halimus*, *M. deserti)* and solvent extract type: dichloromethane or aqueous. The results in Table 2 showed that in both TPC and TFC analyses, the dichloromethane extracts exhibit the highest values, while the aqueous extracts demonstrate the lowest values.

**TABLE 2:** Total phenolic content (TPC), total flavonoid content (TFC), and antioxidant activity (expressed as EC_50_) measured by DPPH, FRAP, and ABTS assays.

Extract	TPC	TFC	DPPH	FRAP	ABTS
	(mg GAE/g extract)	(mg QE/g extract)	EC_50_(mg/L)		
**Md_Dc_**	17.164 ±0.065^b^	26.409 ±0.109^a^	148.367 ±0.008^d^	163.452 ±0.007^b^	41.162 ±0.000^d^
**AH_Dc_**	26.682 ±0.094^a^	12.023 ±0.074^b^	195.091 ±0.004^b^	203.202 ±0.002^a^	51.151 ±0.000^b^
**Md_Aqu_**	5.907 ±0.045^d^	0.408 ±0.003^d^	185.013 ±0.002^c^	144.814 ±0.002^c^	50.487 ±0.001^c^
**AH_Aqu_**	11.794 ±0.069^c^	1.267 ±0.002^c^	199.688 ±0.003^a^	119.405 ±0.000^d^	55.902 ±0.002^a^
**Acid ascorbic**			2.647 ±0.372	2.457 ± 0.167	1.271 ± 0.057

Initially, the dichloromethane extract of *A. halimus* (AH_Dc_) showed a significantly higher level of total phenols content (*p* < 0.001) compared to the aqueous extract (AH_Aqu_), with TPC values equal to 26.682 ± 0.094 and 11.794 ± 0.069 mg GAE g^−^^1^ extract, respectively. On the other hand, AH_Dc_ revealed a TFC of 12.023 ± 0.074 mg QE g^−^^1^ of extract, while the aqueous extract showed a TFC value of 1.267 ± 0.002 mg QE g^−^^1^ of extract. Comparatively, a sample of *A. halimus* collected from the Mascara region (Algeria) indicated a TPC ranging from 9.5 ± 0.009 to 12 ± 0.015 mg GAE g^−^^1^ dry weight, while the TFC ranged from 20 ± 0.007 to 24 ± 0.014 mg QE g^−^^1^ for the aqueous extract [37]. In a separate study [38], our results exceeded those of the ethanolic extract from *A. halimus* samples collected in Setif (Algeria), which reported a TPC of 15.406 ± 0.003 mg GAE g^−^^1^ extract and a TFC of 4.350 ± 0.023 mg QE g^−^^1^extract. In contrast, the aqueous fraction exhibited a higher TPC and TFC values of 19.601 ± 0.016 mg GAE g^−^^1^ extract and 1.995 ± 0.026 mg QE g^−^^1^extract, surpassing our findings. Moreover, a study of *A. halimus* from El Oued and Tlemcen regions in Algeria [39] revealed that the leaves of a sample from El Oued contain a higher TPC (10.25 ± 1.17 mg GAE/g DW), while the Tlemcen samples had a greater TFC (3.09 ± 0.13 mg EC/g DW). Additionally, the highest TPC and TFC values were observed in samples collected from Boukais, South Western Algeria [40], with a TPC of 68.20 mg GAE g^−^^1^ and a TFC of 439 mg QE g^−^^1^ dry butanoic extract. Our TFC results were also higher than those reported in another study [41], which found values of 2.04 mg QE g^−^^1^ in the methanol extract and 1.48 mg QE g^−^^1^ in the aqueous extract. Furthermore, a study of the aerial parts (leaves and stems) of *A. halimus* from three Algerian sites (Mazagran, Biskra, and Sig) showed that extracts from Mazagran leaves and stems were richer in TPC (279.33 ± 1.17 mg GAE/g LE and 68.11 ± 7.76 mg GAE/g LE, respectively) and TFC (98.19 ± 1.51 mg RE/g LE and 64.35 ± 0.41 mg RE/g LE, respectively [42]. Meanwhile, the values of TPC and TFC were 36.29 ± 2.18–33.28 ± 2.06 mg GAE/g DW and 8.11 ± 0.30–4.57 ± 0.45 mg QE/g DW in the aqueous and hydroalcoholic (leaves) extracts, respectively, for a sample collected from Morocco [43].

Therefore, the dichloromethane extract of *M. deserti* (Md_Dc_) demonstrates a TPC of 17.164 ± 0.065 mg GAE g^−^^1^ extract and TFC of 26.409 ± 0.109 mg QE g^−^^1^ of extract, while the aqueous extract (Md_Aqu_) has a TPC of 5.907 ± 0.045 mg GAE g^−^^1^ extract and TFC of 0.408 ± 0.003 mg QE g^−^^1^ of extract, with a significance level of *p* < 0.001. These results surpass those reported by Saad et al. [16], which indicated TPC values of (2.34 ± 1.4 and 2.25 ± 1.1 mg EAG g^−1^ extract) for the diethyl ether extract and (0.28 ± 0.2 and 1.47 ± 0.3 mg EAG g^−1^ extract) for the ether extract from the aerial parts of *M. deserti*, sampled in Biskra and Tamanrasset, respectively. Also, our results surpass those reported by Ghedadba et al. [13], which indicated a TPC of 34 ± 0.43 mg GAE 100 g^−^^1^ in dichloromethane extracts and 171 ± 0.30 mg GAE 100 g^−^^1^ in aqueous extracts from *M. deserti* leaves collected in the Daya‐Mogheul region of Bechar. Additionally, the TFC was recorded at 5.81 ± 0.14 mg QE/100 g in the dichloromethane extract and 26.13 ± 0.63 mg QE/100 g in the aqueous extract. However, Edziri et al. [44] reported an even greater phenolic content in the methanol and butanol extracts of a sample from Tunisia, with a TPC value varying from 118 to 26.6 mg GAE g^−^^1^ extract. However, Bousselsela et al. [45] reported the highest TFC at 28.48 ± 0.40 mg QE g^−1^ extract. Similarly, in a study of the same family of our plant conducted by Nohutcu [46], it was reported that the total phenolic content was 133.18 mg GAE g^−1^ extract, and the total flavonoid content was 8.64 mg QE 100 g^−1^ of *M. vulgare* L. leaves from Van, Türkiye. Likewise, Kabach et al. [47] reported that the TPC was (32.710 ± 3.82 and 60.409 ± 6.63 mg EAG g^−1^ DW) and the TFC was (26.023 ± 2.72 and 33.813 ± 2.81 mg QE g^−1^ DW) for the aqueous and methanolic extracts, respectively, of *M. vulgare L*. leaves collected in Taza, Morocco.

The chemical composition of the dichloromethane and aqueous extracts of *A. halimus* and *M. deserti* was analyzed by UHPLC‐MS/MS (Figures 1, 2, 3, 4), the results are presented in Table 3.

**FIGURE 1:**
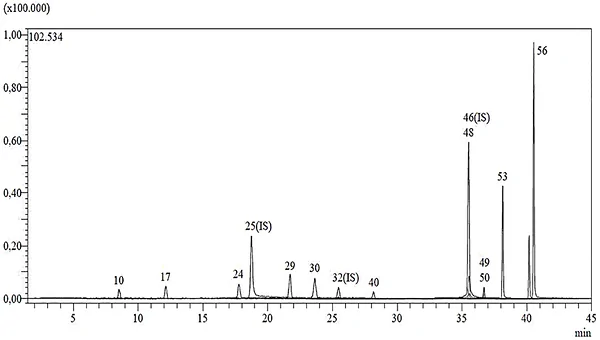
UHPLC/MS‐MS chromatogram of the dichloromethane fraction of *Marrubium deserti* De Noé.

**FIGURE 2:**
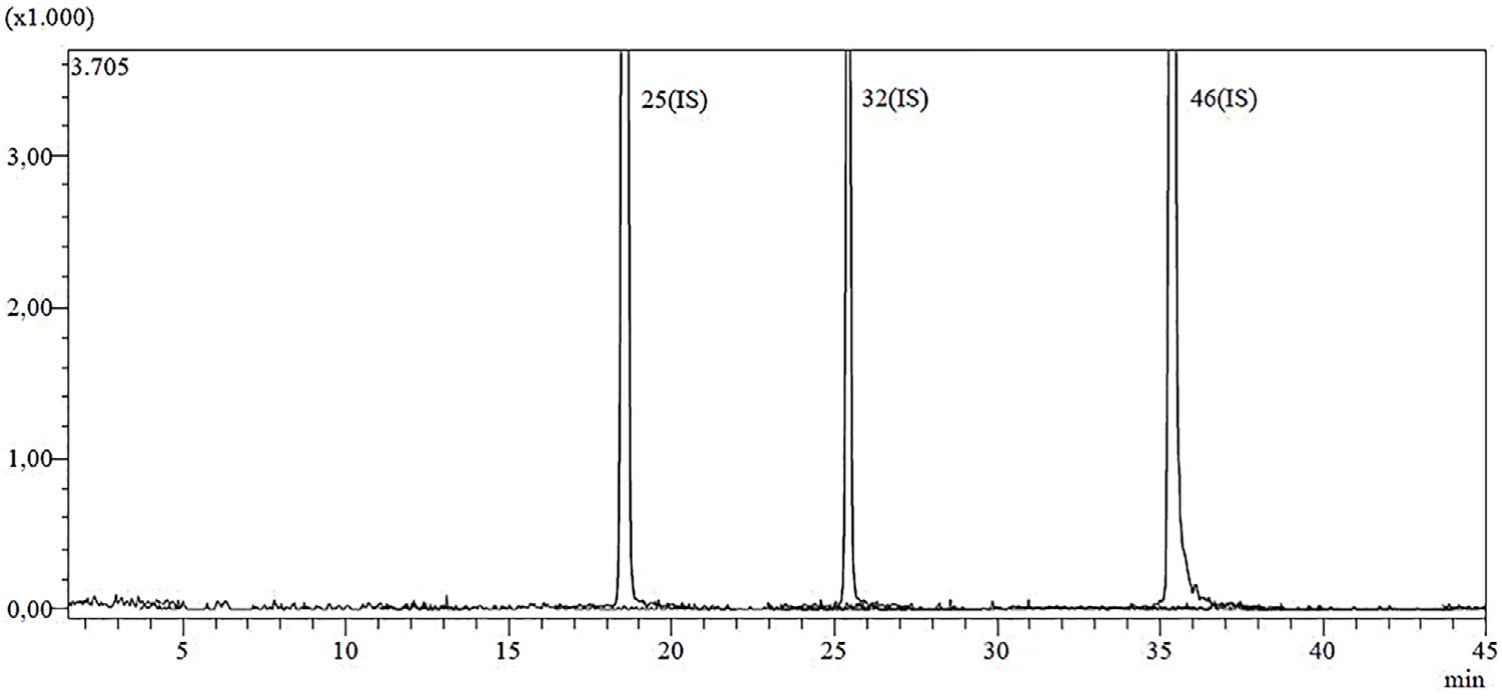
UHPLC/MS‐MS chromatogram of the dichloromethane fraction of *Atriplex halimus* L.

**FIGURE 3:**
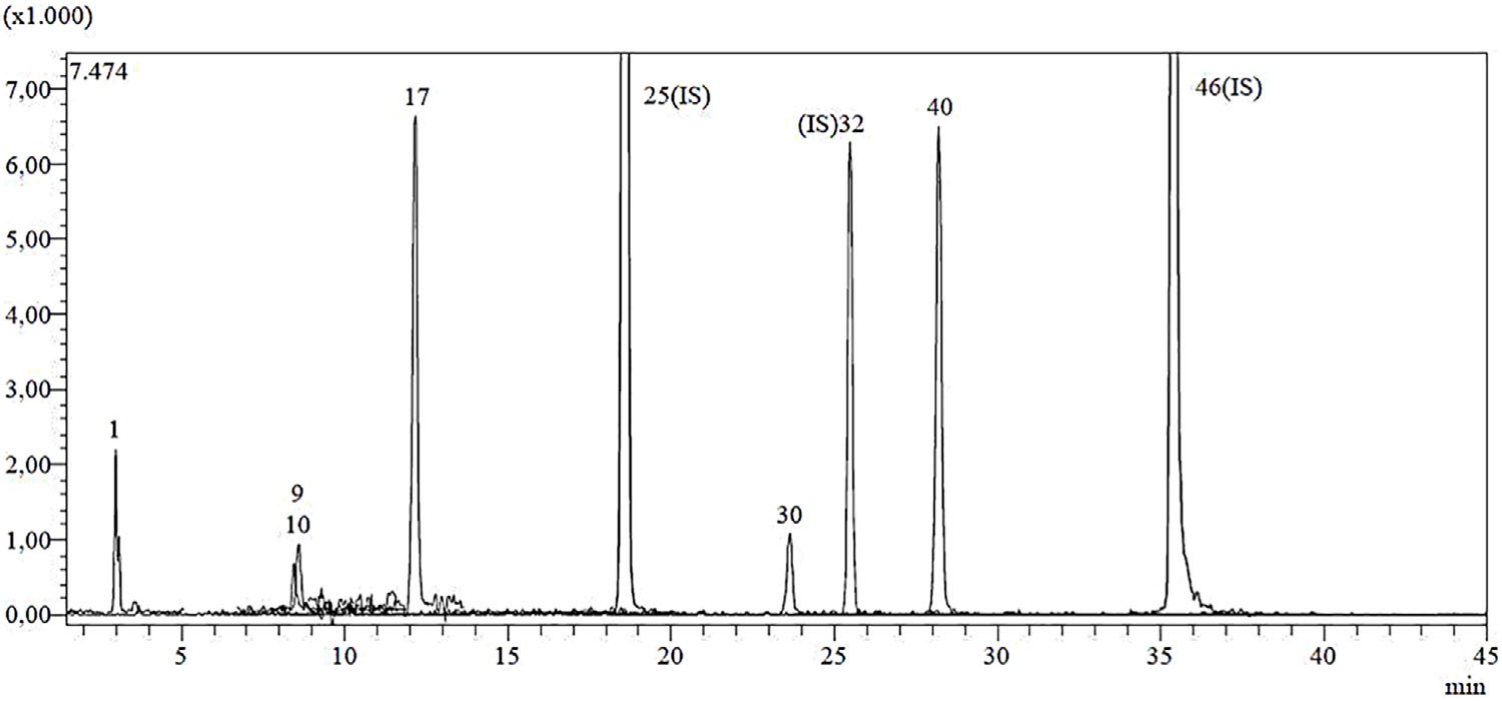
UHPLC/MS‐MS chromatogram of the aqueous fraction of *Marrubium deserti* De Noé.

**FIGURE 4:**
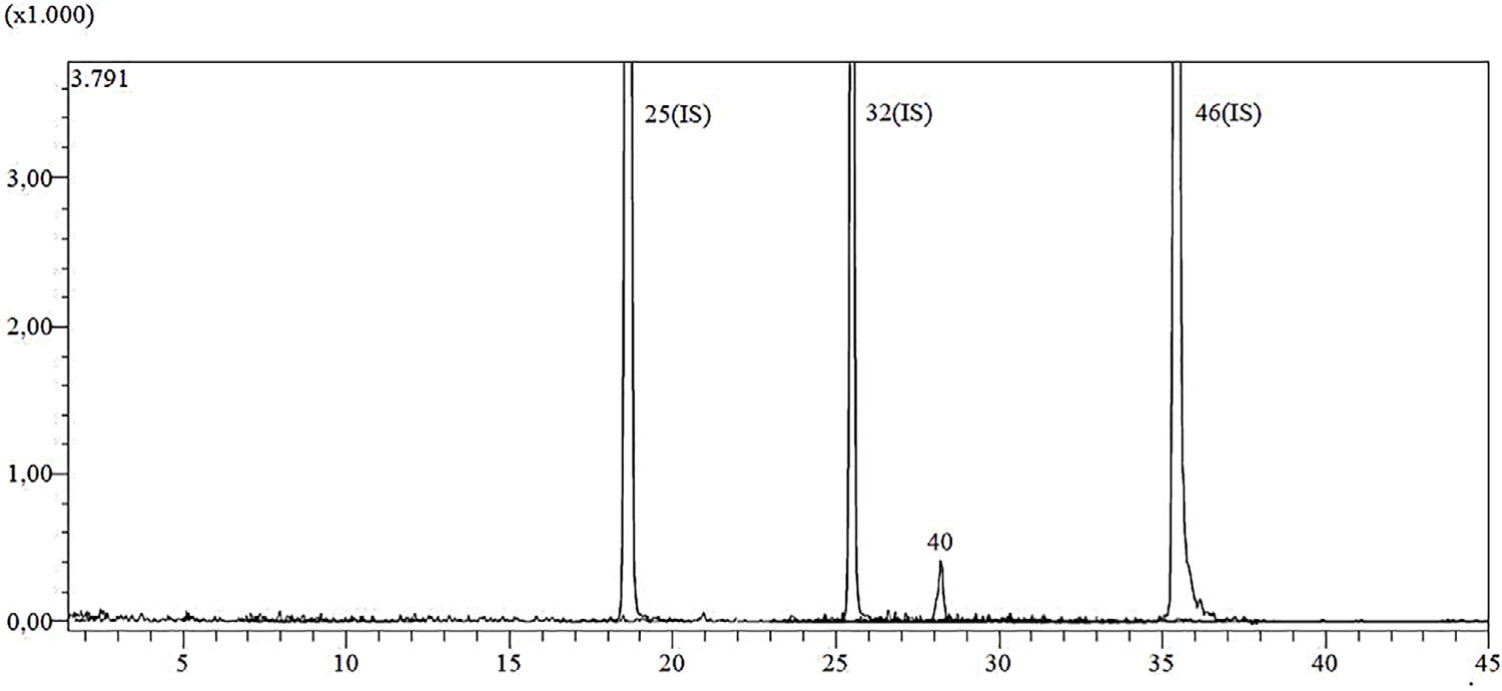
UHPLC/MS‐MS chromatogram of the aqueous fraction of *Atriplex halimus* L.

**TABLE 3:** UHPLC‐MS/MS phenolic profile of *Atriplex halimus* L. and *Marrubium deserti* De Noé extracts.

Analytes	Molecular formula	RT	*m/z*	Ion. mode	Equation	*R^2^*	*RSD*%			Recovery (%)		mg analyte / g of extract			
							Interday	Intraday	*LOD*/*LOQ*	Interday	Intraday	Md_Dc_	AH_Dc_	Md_Aqu_	AH_Aqu_
Quinic acid	C_7_H_12_O_6_	3.0	190.8	Neg	*y* = −0.0129989 + 2.97989*×*	0.996	0.69	0.51	25.7/33.3	1.0011	1.0083	N.D.	N.D.	**262**	N.D.
Chlorogenic acid	C_16_H_18_O_9_	8.4	353.0	Neg	*y* = 0.289983 + 36.3926*×*	0.995	2.15	1.52	13.1/17.6	1.0000	1.0023	N.D.	N.D.	**5**	N.D.
Protocatechuic aldehyde	C_7_H_6_O_3_	8.5	137.2	Neg	*y* = 0.257085 + 25.4657*×*	0.996	2.08	0.57	15.4/22.2	1.0002	0.9988	**13**	N.D.	**16**	N.D.
Caffeic acid	C_9_H_8_O_4_	12.1	179.0	Neg	*y* = 0.120319 + 95.4610*×*	0.999	1.11	1.25	7.7/9.5	1.0015	1.0042	**26**	N.D.	**40**	N.D.
*p*‐Coumaric acid	C_9_H_8_O_3_	17.8	163.0	Neg	*y* = 0.0249034 + 18.5180*×*	0.999	1.92	1.43	25.9/34.9	1.0049	1.0001	**34**	N.D.	N.D.	N.D.
Salicylic acid	C_7_H_6_O_3_	21.8	137.2	Neg	*y* = 0.239287 + 153.659*×*	0.999	1.48	1.18	6.0/8.3	0.9950	0.9998	**42**	N.D.	N.D.	N.D.
Cynaroside	C_21_H_20_O_11_	23.7	447.0	Neg	*y* = 0.280246 + 6.13360*×*	0.997	1.56	1.12	12.1/16.0	1.0072	1.0002	**13**	N.D.	**36**	N.D.
Cosmosiin	C_21_H_20_O_10_	28.2	431.0	Neg	*y* = −0.708662 + 8.62498*×*	0.998	1.65	1.30	6.3/9.2	0.9940	0.9973	**75**	N.D.	**213**	**13**
Naringenin	C_15_H_12_O_5_	35.9	270.9	Neg	*y* = −0.00393403 + 14.6424*×*	0.999	2.34	1.69	2.6/3.9	1.0062	1.0020	**17**	N.D.	N.D.	N.D.
Hesperetin	C_16_H_14_O_6_	36.7	301.0	Neg	*y* = + 0.0442350 + 6.07160*×*	0.999	2.47	2.13	7.1/9.1	0.9998	0.9963	**8**	N.D.	N.D.	N.D.
Luteolin	C_15_H_10_O_6_	36.7	284.8	Neg	*y* = −0.0541723 + 30.7422*×*	0.999	1.67	1.28	2.6/4.1	0.9952	1.0029	**5**	N.D.	N.D.	N.D.
Apigenin	C_15_H_10_O_5_	38.2	268.8	Neg	*y* = 0.119018 + 34.8730*×*	0.998	1.17	0.96	1.3/2.0	0.9985	1.0003	**77**	N.D.	N.D.	N.D.
Acacetin	C_16_H_12_O_5_	40.7	283.0	Neg	*y* = −0.559818 + 163.062*×*	0.997	1.67	1.28	1.5/2.5	0.9949	1.0011	**485**	N.D.	N.D.	N.D.

Abbreviations: *LOD*/*LOQ* (µg/L), limit of detection/quantification; *m/z*, molecular ions of the standard analytes (*m/z* ratio); R.T., retention time; *R^2^*, coefficient of determination; *RSD*, relative standard deviation.

Among these extracts, the *M. deserti* extract contains the highest number of compounds, with 11 identified in the dichloromethane extract (Md_Dc_) and 6 in the aqueous extract (Md_Aqu_). In the Md_Dc_ extract, the following compounds were detected: protocatechuic aldehyde, caffeic acid, p‐coumaric acid, salicylic acid, cynaroside, cosmosiin, naringenin, hesperetin, luteolin, apigenin, and acacetin. Their concentrations were (13, 26, 34, 42, 13, 75, 17, 8, 5, 77, and 485 mg/g of extract), respectively. In the Md_Aqu_ extract, the detected compounds were quinic acid, chlorogenic acid, protocatechuic aldehyde, caffeic acid, cynaroside, and cosmosiin, with concentrations of (262, 5, 16, 40, 13, and 5 mg/g of extract), respectively.

Compared to other studies, most of these compounds have previously been identified in various Marrubium species. Saad, Ouafi, and Chabane [15]. in the aqueous extract of *M. deserti*, For instance, Bursal, Aras, and Kılıç [48] demonstrated that the ethanolic extract of *Marrubium astracanicum subsp. macrodon* contains quinic acid, chlorogenic acid, p‐coumaric acid, salicylic acid, naringenin, and apigenin. Additionally, Ebru Derelli Tüfekçi et al. [49] reported that the main phenolic compounds in methanol–chloroform (1:1) extracts of *M. trachyticum Boiss*. from Turkey were chlorogenic acid, caffeic acid, and luteolin. Furthermore, according to a report by Moshari‐Nasirkandi et al. [50], the HPLC analyses of hydro‐methanolic extracts of 20 various species within the Lamiaceae family showed several compounds, including chlorogenic acid, caffeic acid, p‐coumaric acid, luteolin, and apigenin.

In contrast, for the A. *halimus* extracts, no phenolic compounds were detected in the AH_Dc_ sample, while only one compound (cosmosiin) was identified in the AH_Aqu_ sample at a concentration of 13 mg/g. This may be attributed to the presence of other phytochemicals, such as alkaloids or saponins, which could be responsible for the observed activities but were not detected by HPLC. Nevertheless, previous studies have reported apigenin and naringenin in the HPLC analyses of the aqueous extracts of the aerial parts of *A. halimus* collected in Algeria [51]. Similarly, ethanolic extract of *A. halimus* leaves collected from Morocco showed caffeic acid and chlorogenic acid in its LC‐MS analyses [52]. Moreover, salicylic acid and caffeic acid were detected in the methanolic extract of samples of *A. halimus* collected from Tunisia [53]. In addition, p‐coumaric acid, hesperetin, apigenin, luteolin and apigenin were identified in aqueous, hydroethanolic, and ethanolic extracts of the aerial part of a sample of *A. halimus* collected from Morocco [54].

### 3.2 Antioxidant Activity

Various antioxidant assays are effective for evaluating the antioxidant potential of bioactive compounds in plant extracts. However, relying on a single method cannot capture all the mechanisms underlying antioxidant activity, as plant extracts contain complex mixtures of phytochemicals [55, 56]. The determination of the antioxidant properties of the extract was performed using three methods: ABTS, DPPH, and FRAP assays, and to evaluate the potency of the extracts, ascorbic acid was employed as a positive control. The values of the standard were: 2.647 ± 0.372, 2.457 ± 0.167 and 1.271 ± 0.057 mg/L for the DPPH, ABTS, and FRAP assays, respectively. The results are summarized in Table 2.

The dichloromethane extracts of *M. deserti* (Md_Dc_) demonstrated notable antioxidant properties, as evidenced by their corresponding EC_50_ values. Specifically, the extract exhibited an EC_50_ of 148.367 ± 0.008 mg/L for DPPH, an EC_50_ of 163.452 ± 0.007 mg/L for FRAP and an impressive EC_50_ of 41.162 ± 0.000 mg/L for ABTS. In contrast, the aqueous extract of *M. deserti* presented varying antioxidant capacities. The EC_50_ for DPPH was 185.013 ± 0.002 mg/L, an EC_50_ of 144.814 ± 0.002 mg/L for FRAP and an EC_50_ of 50.487 ± 0.001 mg/L for ABTS. These findings highlight the varying degrees of antioxidant activity present in the different extract types of *M. deserti*. The results we obtained for the dichloromethane extract are significantly better than those reported by Ghedadba et al., who found that the dichloromethane extract exhibited very low antiradical activity in the DPPH assay, with an IC50 of 500 mg/L. But they found a higher antioxidant capacity in the aqueous extract, with an IC_50_ of 125.2 mg/L in the DPPH assay [13]. According to recent research, the methanolic extract of a sample collected from the Daya Mouguel—Bachar showed a strong antioxidant in the DPPH tests, with IC_50_ values of 15.1 mg/L [57]. In another study, the EC_50_ for DPPH and ABTS was recorded at 1.25 ± 0.12 and 13 ± 1.13 mg/mL, respectively, for the chloroform extracts [44]. In addition, the antioxidant activity of other plants in the same family demonstrated good antioxidant capacity. For example, the methanolic extract of *M. vulgare* collected from Saudi Arabia showed significant antioxidant capacity against DPPH (IC_50_ = 35 ± 0.01 mg/L) and ABTS (IC_50_ = 25 ± 0.2 mg/L) [58].

On the other hand, for the extract of *A. halimus*, the dichloromethane extract demonstrates notable efficacy, with EC_50_ values of 195.091 ± 0.004 mg/L in the DPPH assay, 203.202 ± 0.002 mg/L in the FRAP assay, and an impressive 51.151 ± 0.000 mg/L in the ABTS assay. In contrast, the aqueous extract shows slightly higher EC_50_ values in the FRAP assay, with a value of 119.405 ± 0.000 mg/L, while displaying lower values in DPPH and ABTS assays, measuring 199.688 ± 0.003 mg/L in DPPH and 55.902 ± 0.002 mg/L in ABTS.

Our results exceeded those reported for aqueous extracts by Ould Kaddour et al. [51], who investigated the aerial parts of *A. halimus* from three regions in Algeria: Mazagran, Biskra, and Sig. In their study, the IC*50* values for the DPPH assay were reported as follows: 623.684 ± 3.90 mg/L for Biskra stems and 654.165 ± 1.32 mg/L for Mazagran leaves. In the ABTS assay, the values obtained were 355.94 ± 1.59 mg/L for Biskra leaves, 356.58 ± 3.20 mg/L for Mazagran leaves, and 349.75 ± 10.00 mg/L for Sig leaves. Similarly, Roubi et al. [59] demonstrated great results, where they found that the IC_50_ equal to (0.59 ± 0.12 and 1.21 ± 0.09 mg/mL) in DPPH and (39.10 ± 1.62 mg and 67.15 ± 3.19 mg/mL) in ABTS, for the hydroethanolic and the aqueous extracts, respectively. Another study found the best results for the ethanolic leaf extract. In the DPPH assay, with an IC50 of 0.36 ± 0.05 and 27.40 ± 1.46 mg/mL in the FRAP assay, respectively [60]. Moreover, extracts of samples collected in the region of Tunisia demonstrated significant antioxidant activity, as assessed by the DPPH assay IC50 = 147.3 mg/L for the Seliena sample [53]. However, the IC_50_ of DPPH was 520.64 and 193.47 mg/L and the EC_50_ of FRAP was 5 mg/mL for the methanolic–acetonic extract of al Oued and Tlemcen samples, respectively [39].

Generally, the dichloromethane extracts, particularly the AH_Dc_ extract, were consistently more effective than the aqueous extracts, even though their UHPLC–MS/MS phytochemical profile did not reveal any identifiable compounds. This observation suggests that the medium polarity of dichloromethane favors the extraction of bioactive compounds in these species compared to the aqueous solvent. Moreover, these extracts exhibited higher total phenolic content (TPC), as estimated by the Folin–Ciocalteu reagent, which can react with various reducing substances, contributing to the measured values and may be responsible for the observed antioxidant activity.

### 3.3 Antimicrobial Activity

The antimicrobial activity of the extracts against the three bacterial strains and *C. albicans* was given in Table 4.Initially, for the efficacy of the antibiotic, the Gentamicin showed the following results against the *S. aureus*: a MIC_50_ of 0.0013 mg/mL, a MIC_90_ of 0.0036 mg/mL, and a MBC of 0.0042 mg/mL while against the *P. aeruginosa*, the MIC_50_ was 0.017 mg/mL, the MIC_90_ was 0.116 mg/mL, and the MBC was 0.141 mg/mL In the case of *E. coli*, the MIC_50_ was 0.0021 mg/mL, the MIC _90_ was 0.0039 mg/mL, and the MBC was 0.0044 mg/mL In contrast, the antibiotic Imipenem had the following results: against *S. aureus*, the MIC_50_ was 0.0004 mg/mL, MIC_90_ was 0.0008 mg/mL, and MBC was 0.0009 mg/mL; for *P. aeruginosa*, the MIC_50_ was 0.036 mg/mL, MIC_90_ was 0.246 mg/mL, and MBC was 0.299 mg/mL; and for *E. coli*, the MIC_50_ was 0.00095 mg/mL, MIC_90_ was 0.0017 mg/mL, and MBC was 0.0019. For the antifungal agent Fluconazole against *C. albicans*, the results were a MIC_50_ of 0.0003 mg/mL, a MIC_90_ of 0.0005 mg/mL, and an MBC of 0.0006 mg/mL. Nevertheless, when we compared the efficacy of the antibiotic to the extract, we found that the antibiotic outperformed the extract for both *S. aureus* and *E. coli* strains. The antibiotic achieved effectiveness rates ranging from 100 to 12 000 times for *S. aureus* and between 1000 and 5000 times for *E. coli*. In contrast, the extract demonstrated a *pseudo*‐effectiveness rate of only 5 to 80 times. Notably, the effectiveness rate against *C. albicans* was consistently just 10 times higher than that of all extracts. These results indicate that our extract is a potent anti‐*P. aeruginosa* agent and possesses strong antifungal properties.

**TABLE 4:** Results of the antimicrobial activity.

Extract	*Staphylococcus aureus*			*Pseudomonas aeruginosa*			*Escherichia coli*			*Candida albicans*		
	MIC_50%_	MIC_90%_	MBC	MIC_50%_	MIC_90%_	MBC	MIC_50%_	MIC_90%_	MBC	MIC_50%_	MIC_90%_	MFC
	(mg/mL)											
**Md_Dc_**	0.224 ± 0.000^d^	0.397 ± 0.000^d^	0.440 ± 0.000^d^	1.280 ± 0.000^b^	2.282 ± 0.000^b^	2.532 ± 0.000^b^	3.020 ± 0.000^b^	5.438 ± 0.000^b^	6.043 ± 0.000^b^	0.0029 ± 0.000^c^	0.0054 ± 0.000^c^	0.006 ± 0.000^c^
**AH_Dc_**	4.828 ± 0.000^a^	8.693 ± 0.000^a^	9.659 ± 0.000^a^	0.810 ± 0.000^c^	1.477 ± 0.000^c^	1.644 ± 0.000^c^	4.485 ± 0.000^a^	8.079 ± 0.000^a^	8.977 ± 0.000^a^	0.0026 ± 0.000^d^	0.0047 ± 0.000^d^	0.0053 ± 0.000^d^
**Md_Aqu_**	2.994 ± 0.000^c^	5.706 ± 0.000^c^	6.384 ± 0.000^c^	0.714 ± 0.000^d^	1.272 ± 0.000^d^	1.412 ± 0.000^d^	2.985 ± 0.000^c^	5.404 ± 0.000^c^	6.009 ± 0.000^c^	0.0031 ± 0.000^a^	0.0056 ± 0.000^a^	0.0062 ± 0.000^a^
**AH_Aqu_**	3.307 ± 0.000^b^	5.953 ± 0.000^b^	6.615 ± 0.000^b^	1.337 ± 0.000^a^	2.378 ± 0.000^a^	2.638 ± 0.000^a^	2.588 ± 0.000^d^	4.635 ± 0.000^d^	5.146 ± 0.000^d^	0.0031 ± 0.000^b^	0.0055 ± 0.000^b^	0.0062 ± 0.000^b^
**Gentamicin**	0.0013 ± 0.000	0.0036 ± 0.000	0.0042 ± 0.000	0.017±0.000	0.116 ± 0.000	0.141 ± 0.000	0.0021 ± 0.000	0.0039 ± 0.000	0.0044 ± 0.000		/	
**Imipenem**	0.0004 ± 0.000	0.0008 ± 0.000	0.0009 ± 0.000	0.036 ± 0.000	0.246 ± 0.000	0.299 ± 0.000	0.00095 ± 0.000	0.0017 ± 0.000	0.0019 ± 0.000			
**Fluconazole**	/									0.0003 ± 0.001	0.0005 ± 0.001	0.0006 ± 0.002

Abbreviations: MBC, minimum bactericidal concentration; MFC, minimum fungicidal concentration; MIC_50%_, the minimum inhibitory concentration at 50%; MIC_90%_, the minimum inhibitory concentration at 90%.

Firstly, for the extract of *Marrubium déserti*, the Md_Dc_ extract showed the results as follows: for *S. aureus* strain, the MIC_50_ was 0.224 mg/mL, MIC_90_ was 0.397 mg/mL, and MBC was 0.440 mg/mL; for *P. aeruginosa* strain, the MIC_50_ was 1.280 mg/mL, MIC_90_ was 2.282 mg/mL, and MBC was 2.532 mg/mL; for *E.coli*, the MIC_50_ was 3.020 mg/mL, MIC_90_ was 5.438 mg/mL, and MBC was 6.043 mg/mL; and for *C. albicans*, the MIC_50_ was 0.0029 mg/mL, MIC_90_ was 0.0054 mg/mL, and MBC was 0.006. On the other hand, for the aqueous extract, the MIC_50_ values were 2.994, 0.714, 2.985, and 0.0031 mg/mL, and the MIC_90_ values were 5.706, 1.272, 5.404, and 0.0056 mg/mL The MBC values were 6.384, 1.412, 6.009, and 0.0062 mg/mL for *S. aureus* and *P. aeruginosa*, *E. coli and C. albicans*, respectively.

In a study published by Chemsa et al. [14], the methanolic extract of *M. deserti* growing in the North fringe of the Algerian Sahara showed an MIC of 6.25 and 12.5 mg/mL for *S. aureus* ATCC 25923 and *C. albicans* ATCC 10239, respectively. Furthermore, Ghedadba et al. [13] demonstrated that the dichloromethane and methanolic extracts of leaves of *M. deserti* collected from Daya‐Mogheul, Bechar (Algeria) have an MIC of 1000 and 500 mg/mL, respectively, for all the strains *S. aureus* ATCC 25923*, P. aeruginosa* ATCC 27853*, E. coli* ATCC 25922, and *C. albicans*. Recent research on family of *Marrubium* plant was conducted by Mulondo et al. [61]. *M. vulgare* hydro‐ethanolic extract had an MIC of 1.87 mg/mL against *S. aureus*, 2.5 mg/mL against *E. coli* and 1.75 mg/mL for *C. albicans*. in addition, Previous studies of the methanolic extract of *M. vulgare* harvested from King Saudi Arabia showed an MIC of 12.5 mg/mL and MBC of 25 mg/mL for *E. coli* (ATCC8739) and *P. aeruginosa* (ATCC27853) strain and an MIC of 25 mg/mL and MBC of 50 mg/mL for *S. aureus* strain [58]. Another study showed that the MIC value was 25 mg/mL for both *E. coli (*ATCC11775) and *P. aeruginosa* ATCC 9027, and 6.25 to 12.5 for *C. albicans ATCC 10231*, and the MBC value was between 25 and 50 mg/mL for the three strains [62].

Secondly, for the extract of *A. halimus*, the AH_Dc_ extract showed the results as follows: for *S. aureus* strain, the MIC_50_ was 4.828 mg/mL, MIC_90_ was 8.693 mg/mL, and MBC was 9.659 mg/mL; for *P. aeruginosa* strain, the MIC_50_ was 0.810 mg/mL, MIC_90_ was 1.477 mg/mL and MBC was 1.644 mg/mL; for *E. coli*, the MIC_50_ was 4.485 mg/mL, MIC_90_ was 8.079 mg/mL, and MBC was 8.977 mg/mL; and for *C. albicans*, the MIC _50_ was 0.0026 mg/mL, MIC_90_ was 0.0047 mg/mL, and MBC was 0.0053 mg/mL. While the aqueous extract demonstrated the following results: MIC_50_: 3.307 mg/mL (*S. aureus*), 1.337 mg/mL (*P. aeruginosa*), 2.588 mg/mL (*E. coli)*, 0.0031 mg/mL (*C. albicans)* and MIC_90_: 5.953 mg/mL (*S. aureus)*, 2.378 mg/mL (*P. aeruginosa*), 4.635 mg/mL (*E. coli*), 0.0055 mg/mL (*C. albicans*) and MBC: 6.615 mg/mL (*S. aureus)*, 2.638 mg/mL (*P. aeruginosa*), 5.146 mg/mL (*E. coli*), 0.0062 mg/mL *(C. albicans)*.

However, in a study of the methanolic–acetonic extract of a sample of *A. halimus* collected from El Oued‐Algeria, the MIC was 2.5 mg/mL for *S. aureus* ATCC 6538 [39]. Meanwhile, the chloroform and 70% aqueous ethanol extracts of *Atriplex lindleyi Moq*. from Egypt, showed antimicrobial activity against *S. aureus, P. aeruginosa*, and *E. coli* with MIC values of 62 to 125 × 10^3^ mg/mL and MBC values of 125 to 500 ×10^3^ mg/mL. They also had antifungal effects against *C. albicans* with an MIC of 62.5 ×10^3^ mg/mL [63].In comparison to the results obtained by Abdoulaye et al. [64]. who studied the methanol extract from the leaves of *Atriplex cruentus*, which belongs to the same family as *A. halimus*, the effectiveness against strains of *S. aureus* ATCC 25923, *E. coli* ATCC 25922, and *P. aeruginosa* ATCC 27853 exhibited MIC values greater than 30 mg/mL. Moreover, the antimicrobial effects of three varieties of *Atriplex tricolor* leaves showed varying activity against different microorganisms. They were effective against Gram‐positive *S. aureus* ATCC 25923 (MIC: 4‐32 mg/mL), Gram‐negative *P. aeruginosa* ATCC 27853 (MIC: 4‐32 mg/mL), and the yeast *C. albicans* ATCC 10231 (MIC: 8‐32 mg/mL) [65].

### 3.4 Computational Analysis

The in silico assessment of 13 phenolic compounds, as illustrated in Figure 5 by the chemical structures derived from *M. deserti* and *A. halimus*, reveals a complex correlation among predicted biological spectra (PASS), pharmacokinetic profiles (ADMET), and molecular docking affinities.

**FIGURE 5:**
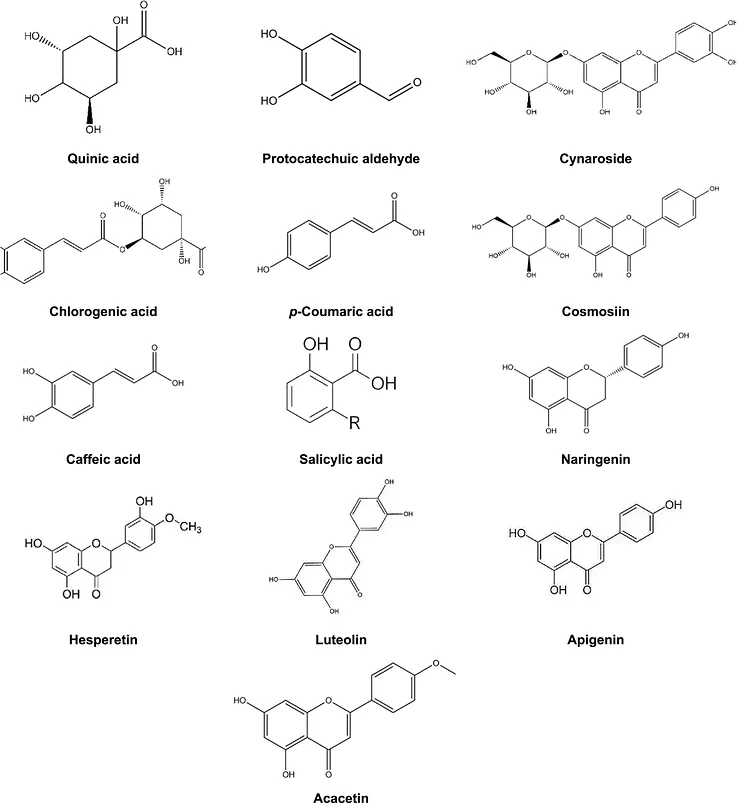
Structural elucidation of compounds identified in the extracts.

The biological activity prediction (P_a_ and P_i_) across antioxidant, antifungal, and antimicrobial domains (Table 5), highlights a clear superiority of flavonoid glycosides and caffeoylquinic derivatives over their respective aglycones and simple phenolic acids. Excluding the standard references Ascorbic acid (P_a_ = 0.928), Gentamicin (P_a_ = 0.831), and Fluconazole (P_a_ = 0.726), the overall highest predicted activities are observed in the C‐7 glycosylated flavones cynaroside (luteolin‐7‐O‐glucoside; P_a_ = 0.841 antioxidant, P_a_ = 0.721 antifungal, P_a_ = 0.597 antimicrobial) and cosmosiin (apigenin‐7‐O‐glucoside; P_a_ = 0.831 antioxidant, P_a_ = 0.729 antifungal, P_a_ = 0.597 antimicrobial), followed closely by chlorogenic acid (P_a_ = 0.785 antioxidant, P_a_ = 0.638 antifungal) and quinic acid (P_a_ = 0.830 antioxidant). In contrast, aglycones like luteolin (P_a_ = 0.775 antioxidant, P_a_ = 0.520 antifungal, P_a_ = 0.388 antimicrobial), apigenin (P_a_ = 0.732 antioxidant, P_a_ = 0.524 antifungal, P_a_ = 0.391 antimicrobial), and naringenin (P_a_ = 0.794 antioxidant, P_a_ = 0.594 antifungal), along with simple phenolic acids like caffeic acid (P_a_ = 0.603), p‐coumaric acid (P_a_ = 0.553), protocatechuic aldehyde (P_a_ = 0.443), and salicylic acid (P_a_ = 0.318), yield lower overall active probabilities. This structural–activity analysis demonstrates that while B‐ring catechol hydroxylation (3',4'‐OH in luteolin, 4'‐OH in apigenin and caffeic, p‐coumaric acid) enhances radical scavenging potential via stabilized o‐quinone formation, O‐glycosylation at the C‐7 position represents the primary structural driver for boosting multi‐target efficacy increasing antioxidant capacity by 8.5%–13.5%, antifungal activity by ∼39%, and antimicrobial potential by >52%–53% compared to corresponding aglycones due to improved hydrophilic solubility, cell membrane interaction kinetics, and cellular transport mechanisms; conversely, O‐methylation like acacetin; O‐glycosylation at the C‐7 = 0.628 attenuates electron‐donating performance, C2═C3 ring‐C saturation such as naringenin offers minor flexibility advantages for fungal membrane disruption, and esterification of caffeic acid with quinic acid into chlorogenic acid significantly expands biological binding sites and functional activity. Pointedly, PASS predictions represent structure–activity relationship probabilities rather than quantitative biological determinations. High Pa values indicate a high structural probability of activity but do not account for dosage, extract matrix dynamics, or physiological stability. Consequently, these in silico spectra serve strictly as preliminary hypotheses that are validated by our experimental in vitro antimicrobial and antioxidant assay results.

**TABLE 5:** Predicted biological activity (PASS) results.

	Antioxidant		Antifungal		Antimicrobial	
Compounds	Pa	Pi	Pa	Pi	Pa	Pi
Acacetin	0.628	0.004	0.511	0.029	0.367	0.039
Apigenin	0.732	0.004	0.524	0.027	0.391	0.032
Caffeic acid	0.603	0.005	0.450	0.039	0.358	0.041
Chlorogenic acid	0.785	0.004	0.638	0.014	0.537	0.013
Cosmosiin	0.831	0.003	0.729	0.008	0.597	0.009
Cynaroside	0.841	0.003	0.721	0.009	0.597	0.009
Hesperetin	0.746	0.004	0.562	0.022	0.370	0.038
Luteolin	0.775	0.004	0.520	0.027	0.388	0.033
Naringenin	0.794	0.003	0.594	0.019	0.397	0.031
p_Coumaric acid	0.553	0.005	0.451	0.039	0.343	0.045
Protocatechuic aldehyde	0.443	0.009	0.480	0.034	0.389	0.033
Quinic acid	0.830	0.003	0.499	0.031	0.478	0.019
Salicylic acid	0.318	0.020	0.395	0.051	0.404	0.029
Ascorbic acid	0.928	0.003	/	/	/	**/**
Gentamicin	/	/	/	/	0.831	0.002
Imipenem	/	/	/	/	0.627	0.007
Fluconazol	/	/	0.726	0.008	/	**/**

Pa = probability to be active; Pi = probability to be inactive.

To systematically evaluate the drug‐likeness and pharmacokinetic profiles of the identified constituents, in silico ADMET properties were predicted (Table 6). A key determinant of oral bioavailability is gastrointestinal absorption, governed by molecular weight, Lipinski parameters, and topological polar surface area (TPSA). Small lipophilic aglycones such as p‐coumaric acid, apigenin, and naringenin exhibited human intestinal absorption (HIA) values exceeding 90%, indicating excellent passive membrane permeability and high predicted oral bioavailability. Conversely, highly polar, hydrophilic glycosides, and ester derivatives, notably chlorogenic acid (HIA 11.2%) and quinic acid (HIA 12.6%) demonstrated poor intestinal absorption, likely requiring active transporter mechanisms or pre‐systemic hydrolytic cleavage by intestinal microbiota to yield bioavailable aglycones. Distribution parameters revealed distinct tissue‐partitioning behaviors: acacetin exhibited the highest volume of distribution at steady state VD_SS_ = 1.559 log L/kg and central nervous system penetration (log BB = 0.222), owing to its low TPSA and favorable lipophilicity. In contrast, polar glycosides like cosmosiin, cynaroside, and complex hydroxycinnamic acids (chlorogenic acid) were restricted from crossing BBB. Metabolic and clearance profiling indicated that small phenolic acids, such as protocatechuic aldehyde and caffeic acid, undergo rapid systemic elimination, exhibiting the highest total clearance values (17.12 and 14.36 mL/min/kg, respectively). From a drug‐interaction perspective, while most compounds spared major cytochrome P450 enzymes, key flavonoids including apigenin, hesperetin, luteolin, and naringenin were identified as potential CYP3A4 inhibitors, with luteolin also demonstrating CYP2D6 inhibition. This highlights potential herb–drug interaction risks if these flavonoids achieve high systemic concentrations. Acute and chronic toxicity predictions categorized cosmosiin and chlorogenic acid as highly tolerable (LD_50_ > 2.6 mol/kg. LOAEL > 4.0 log mg/kg/day), whereas phenolic aldehydes displayed lower safety margins (LD_50_ < 2.0 mol/kg).

**TABLE 6:** Predicted pharmacokinetic properties of identified phenolic compounds.

Property	Compound	Acacetin	Apigenin	Caffeic _acid	Chlorogenic _acid	Cosmosiin	Cynaroside	Hesperetin	Luteolin	Naringenin	p_Coumaric _acid	Protocatechuic _aldehyde	Quinic_ acid	Salicylic _acid
**Absorption**	**Water solubility**	−2.925	−2.989	−1.565	−2.677	−3.527	−3.228	−3.497	−3.197	−3.278	−1.52	−0.901	0.058	−1.662
	**Caco‐2**	−0.229	0.917	−0.012	0.265	0.087	0.169	0.466	1.005	1.166	1.205	1.136	−0.494	1.13
	**HIA**	77.207	91.502	55.525	11.223	39.657	36.734	83.555	84.492	90.508	93.924	71.061	12.58	77.71
	**SP**	−2.735	−2.805	−2.704	−2.737	−2.735	−2.735	−3.672	−2.736	−3.225	−2.793	−2.608	−3.008	−2.322
**Distribution**	**VDss**	1.559	−0.177	−0.559	−0.153	−0.315	−0.032	−0.804	0.014	−0.029	−0.726	−0.25	−0.58	−0.473
	**BBB**	0.222	−0.957	−0.836	−1.599	−1.469	−1.65	−1.013	−1.171	−0.969	−0.23	−0.275	−1.165	−0.313
	**PPB**	97.58	96.53	64.66	64.83	83.75	81.07	97.07	97.64	94.98	68.31	42.03	14.84	85.64
	**CNS**	−2.735	−2.176	−3.36	−4.338	−4.171	−4.48	−2.964	−2.465	−2.236	−2.409	−2.901	−5.098	−2.909
**Metabolism**	**CYP3A4 substrate**	No	No	No	No	No	No	yes	No	No	No	No	No	No
	**CYP3A4 inhibitor**	yes	yes	No	No	No	No	yes	Yes	yes	No	yes	No	No
	**CYP2C19 inhibitor**	No	No	No	No	No	No	No	No	No	No	No	No	No
	**CYP2C9 inhibitor**	No	No	No	No	No	No	No	No	No	No	No	No	No
	**CYP2D6 inhibitor**	No	No	No	No	No	No	No	Yes	No	No	No	No	No
**Excretion**	**TC**	0.407	0.693	0.582	0.402	0.72	−4.48	0.03	0.687	0.068	0.696	0.591	0.68	0.655
	**CL**	5.282	5.939	14.357	3.339	3.213	3.646	4.432	8.481	6.893	7.547	17.116	1.659	2.667
**Toxicity**	**OralRat AcuteTox. (LD50)**	2.471	2.327	1.98	3.19	2.694	2.432	2.221	2.159	2.189	2.07	1.818	1.963	2.048
	**OralRat ChronicTox.‐LOAEL**	2.612	1.671	1.961	3.173	4.462	4.383	1.988	2.265	1.994	2.841	2.972	2.94	2.213

Water solubility (log mol/L), Caco‐2 (log Papp in 10‐6 cm/s), HIA %, SP (log Kp), VDss (log L/kg), BBB (log BB), PPB (%), CNS (log PS), TC (log mL/min/kg), CL (mL/min/kg), OralRat AcuteTox. (LD_50_) mol/kg), OralRat ChronicTox. ‐LOAEL (log mg/kg_bw/day).

Importantly, these in silico ADMET models evaluate isolated compounds under ideal physiological conditions and do not reflect the complexity of crude *A. halimus* and *M. deserti* extracts. In crude matrices, co‐occurring phytochemicals can alter solubility, permeability, and CYP450/P‐glycoprotein activity, while gut microbiota actively metabolize poorly absorbed compounds like chlorogenic acid into bioavailable metabolites prior to systemic entry. Furthermore, actual in vivo exposure remains constrained by the native concentrations of individual constituents within the extract, highlighting the need for future bioanalytical pharmacokinetic validation.

Molecular docking simulations were conducted to elucidate the structural basis of the observed biological activities. The evaluation included extracts and standard controls: ascorbic acid for antioxidant potential, and gentamicin, imipenem, and fluconazole for antimicrobial efficacy. These were tested against six molecular targets selected for their biological relevance to the pathogens and oxidative stress pathways examined. MecR1, LpxC, and GyrB24 are important proteins in *S. aureus, E. coli*, and *P. aeruginosa*. Fur1p represents antifungal targets. AKR1B1, Nrf2, and FOL are oxidative stress mediators in host redox balance. Table 7 and Figures 6, 7, 8, 9, 10, 11, 12 characterize the specific binding affinities underlying these bioactivities.

**TABLE 7:** The results of the docking study.

Compose	Binding energy (Kcal/Mol)	Closest residues	Hydrophobic interactions	Hydrogen bonds	Distance (Å)
**6o9s**					
Acacetin	−7.1	Phe423, Ile533	Pi‐Pi T‐shaped, Alkyl	Trp426	2.75
Apigenin	−7.9	Phe423	Pi‐Pi Stacked	Glu538	1.95
				Thr531	2.32
Caffeic‐acid	−5.7	Phe 423	Pi‐Alkyl	Tyr 438	2.4
				Trp426	2.39
Chlorogenic‐acid	−7.6	Phe423	Pi–Pi Stacked	Trp426	2.42
				Tyr438	2.27
				Glu569	2.28
				Gly568	2.99
Cosmosiin	−8.4	Phe423	Unfavorable donor–donor Pi–Pi T‐shaped	Tyr438	2.17
				Thr529	2.37
				Glu538	2.61
				Trp 426	2.84
Cynaroside	−8.1	Phe423	Unfavorable donor–donor Pi–Pi T‐shaped	Trp426	2.94
				Thr529	2.37
				Lys437	1.87
				Glu538	2.28
				Gln572	2.58
Hesperetin	−7.3	Phe423, Ile533	Pi‐Sigma, Alkyl	Trp426	2.73
Luteolin	−7.1	Leu517, Lys437	Pi‐Sigma	Thr529	2.43
Naringenin	−7	Trp426	Unfavorable donor–donor	Trp426	2.74
				Tyr438	2.4
p‐Coumaric‐acid	−5.5	Phe423	Pi–Pi stacked	Yr438	2.54
Protocatechuic‐aldehyde	−5.2	Phe423	Pi–Pi stacked	Ser439	3.09
				Trp426	2.4
				Tyr438	2.4
Quinic‐acid	−5.5	—	—	Phe423	3.2
				Glu425	2.51
Salicylic‐acid	−4.9	Thr529	Unfavorable donor–donor	Gly568	2.13
				Glu569	2.13
Gentamicin	−7.8	Lys528, Leu517,Trp426,Phe423	Pi‐Sigma, Alkyl, Pi‐Alkyl	Tyr438	3.61
				Ser514	1.98
				Lys437	3.68
				Gln572	2.74
				Thr529	2.82
Imipenem	−6.3	Lys528	Alkyl	Gly527	2.26
				Thr529	2.27
				Met515	3.8
				Lys528	2.42
				Ser439	3.09
**6dui**					
Acacetin	−5.2	Arg201, Gly208	Pi‐cation, pi‐sigma	Gly21	1.89
				Ser210	2.98
Apigenin	−7.7	Met62, Asp196, Phe160	pi‐cation, pi‐anion, pi‐sulfur, pi–pi T‐shaped	Gly192	3.65
Caffeic‐acid	−5.6	—	—	Lys238	2.53
				Phe193	2.33
				Asp196	2.37
Chlorogenic‐acid	−7.7	Met62	Alkyl	Thr61	2.62
				Gly192	3.2
				Phe193	2.23
				Phe191	2.47
				Ser262	2.79
				Lys238	2.52
Cosmosiin	−7.9	Asp196, Met62, Leu200,	pi‐cation, pi‐Anion, pi‐Alkyl, pi–pi T‐shaped	Lys261	2.11
				Tyr199	2.93
Cynaroside	−6.8	Asp196, Met62	pi‐cation, pi‐Anion, pi‐sulfur	Lys238	2.49
				Ser262	2.56
Hesperetin	−7.7	Phe160, Ile158, Leu200, Met62	pi‐Anion, pi‐sulfur, pi‐Alkyl, Alkyl	Asp196	3.06
				Ser262	2.16
				Gly192	3.23
Luteolin	−7.4	Asp196, Met62, Phe160	pi‐Anion, pi‐sulfur, pi‐Alkyl	—	—
Naringenin	−7.8	Asp196, Met62	pi‐cation, pi‐Anion, pi‐Alkyl	—	—
p‐Coumaric‐acid	−6.1	Asp196, Met62, Phe193	pi‐cation, pi‐Anion, pi‐Alkyl, pi–pi T‐shaped	—	—
Protocatechuic‐aldehyde	−5.8	Asp196, Met62	pi‐cation, pi‐Anion, pi‐Alkyl	Phe191	2.38
Quinic‐acid	−6.1	—	—	His264	2.44
				Ala265	1.93
				Thr61	2.8
Salicylic‐acid	−6.3	Asp196	unfavorable acceptor–acceptor, pi–pi T‐shaped	Gly192	2.21
				Phe193	3.42
Gentamicin	−7.2	Leu200, Phe193, Phe160	pi‐Alkyl, alkyl	Lys261	2.44
				Asp159	2.21
Imipenem	−7	Met62, Phe160, Ala265, His264	pi‐sulfur, pi‐Alkyl, alkyl	Thr61	1.97
				Gly263	3.17
				Lys238	2.1
				Asp196	3.71
**7p2m**					
Acacetin	−7.4	Asp49, Arg76, Val120, Ala53	Pi‐Anion, Pi‐Cation, Alkyl, Pi‐Alkyl	Ile94	3.54
				Glu50	2.19
Apigenin	−7.3	Ile94	Pi‐Alkyl	Asp49	2.4
Caffeic‐acid	−6.4	Ile94	Pi‐Sigma	Ser121	2.99
				Val120	2.49
Chlorogenic‐acid	−8.6	Ile94	Unfavorable acceptor–acceptor	Arg76	2.21
				Glu50	2.5
				Leu98	3.09
				Val97	2.52
Cosmosiin	−7.2	Ile94	Pi‐Sigma	Arg76	2.36
				Ile94	2.07
	−8.4	Glu50, Ile94	Unfavorable acceptor–acceptor, pi‐alkyl	Asp49	2.47
				Ser121	2.77
				Val120	2.67
Cynaroside				Arg76	2.64
				Asn46	2.7
Hesperetin	−7.1	Ile94, Glu50, Arg76	Alkyl, Unfavorable acceptor–acceptor, Pi‐Alkyl, Pi‐Cation	Asp49	2.25
Luteolin	−7.2	Arg76	Unfavorable donor–donor	Asn46	2.98
				Ile94	2.52
				Glu50	2.11
				Val120	2.39
Naringenin	−7.1	Asp49, Ile94, Ser121	Unfavorable donor–donor, Pi‐Sigma, Pi‐Anion	—	—
p‐Coumaric‐acid	−6	Ile94	Pi‐Alkyl	Ser121	2.16
				Val120	2.99
				Ile94	2.44
Protocatechuic‐aldehyde	−5	—	—	Arg76	2.15
				Asn46	2.43
Quinic‐acid	−5.4	—	—	Glu50	2.52
				Asn46	1.94
Salicylic‐acid	−5.3	Ile94, Asn46	Pi‐Alkyl, Unfavorable acceptor–acceptor	—	—
Gentamicin	−6.8	Ile94, Ala53	unfavorable acceptor–acceptor, alkyl	Glu50	2.73
				Asn46	3.65
				Gly119	3.61
				Val93	3.51
Imipenem	−5.7	Ile94	alkyl	Arg76	3.04
				Asp49	3.17
				Glu50	3.08
				Val120	2.69
				Gly119	3.77
**7rh8**					
Acacetin	−5.5	Phe76 Interactions	Pi–Pi stacked, Pi‐Alkyl	—	—
Apigenin	−7.6	Phe62	Pi–Pi stacked	—	—
Caffeic‐acid	−4.4	—	—	Lys75	2.95
Chlorogenic‐acid	−5.8	Gly64	Unfavorable acceptor–acceptor	Ser87	2.06
Cosmosiin	−6.2	Lys65	Pi‐Alkyl	Arg86	2.07
Cynaroside	−6.5	Lys65	Pi‐Alkyl	Arg144	2.63
				Glu116	2.85
Hesperetin	−5.7	Lys65	Alkyl, Pi‐Alkyl, Pi–Pi T‐shaped	Lys61	2.01
				Lue63	3.44
				Phe62	2.87
Luteolin	−5.9	Phe62	Pi‐Alkyl, Pi–Pi T‐shaped	Lys61	2.17
				Gly64	2.71
Naringenin	−5.4	Phe62	Pi–Pi stacked	—	—
p‐Coumaric‐acid	−4.2	—	—	Ala60	2.51
				Lys61	2.87
Protocatechuic‐aldehyde	−4.1	Pro111, Arg89	Pi‐Alkyl	Ile90	1.79
				Pro111	3.45
				Arg89	2.73
Quinic‐acid	−4.4	Phe62, Leu42	Alkyl, Pi‐Alkyl	Cyc85	2.6
				Glu40	2.61
Salicylic‐acid	−3.9	Arg86	Pi‐Alkyl	Cyc85	2.89
				Arg86	2.15
Fluconazol	/	Leu56, Leu53, Glu50	Pi‐Anion, Pi‐Alkyl, Halogen (Fluorine)	Glu50	2.34
**8fh8**					
Acacetin	−7.4	Val47, Trp111, Trp79, Phe122	Pi‐Sigma, Alkyl	Trp20	2.45
Apigenin	−7.8	Val47	Pi‐Sigma, pi‐Alkyl	Trp21	2.39
				Gln49	2.08
Caffeic‐acid	−6.5	Val47	Pi‐Sigma	Trp111	2.03
Chlorogenic‐acid	−7.1	Pro215	Alkyl	Lys221	2.34
				Arg217	2.23
				Pro225	2.36
				Arg268	2.13
Cosmosiin	−7.5	Asp216	Pi‐Anion, Unfavorable donor–donor, Pi‐Alkyl	Lys21	2.72
				Ala220	2.8
				Glu229	2.36
				Ser226	2
Cynaroside	−8	Pro225, Pro215, Glu229, Leu228	Pi‐Anion, Unfavorable acceptor–acceptor, Pi‐Alkyl	Thr244	2.93
				Ser226	2.72
				Arg217	2.46
				Lys221	2.12
				Arg268	2.62
Hesperetin	−7	Pro23, Lys21, Val47	pi‐sigma, pi–pi T‐shaped, Alkyl	Trp20	2.29
				Ser22	3.05
				Tyr48	2.69
Luteolin	−7.3	Val47	Pi‐Alkyl, Alkyl	Trp20	2.69
Naringenin	−6.6	Val47	pi‐sigma	Trp2	2.56
				His110	3.09
p‐Coumaric‐acid	−6.2	Trp111, Val47	Unfavorable donor–donor	—	—
Protocatechuic‐aldehyde	−5.3	—	—	Lys221	2.14
				Arg217	2.2
				Pro225	2.01
				Ser226	2.6
Quinic‐acid	−5.5	Arg217	Unfavorable donor–donor	Pro215	1.95
				Pro222	3.65
				Arg217	2.6
				Pro225	2.57
Salicylic‐acid	−5.4	Glu229	pi‐Anion	Lys221	2.14
				Arg217	1.69
				Pro225	2.05
Ascorbic acid	−5.1	Arg217	Unfavorable donor–donor	Lys221	2.19
				Arg217	2.59
				Ser226	2
**5sss**					
Acacetin	−8.2	Met20, Trp22, Leu28	Pi‐Alkyl, Alkyl, pi‐sigma	Leu24	3.19
				Ile50	2.06
				Ser47	2.76
				Asp27	3.74
Apigenin	−8.2	Leu28, Met20	Pi‐Alkyl, pi‐sigma	Ile50	2.01
				Ser49	2.56
Caffeic‐acid	−7.7	Met20	Alkyl	Met20	2.22
				Trp22	2.54
				Leu24	2.11
Chlorogenic‐acid	−8	Leu28	Unfavorable acceptor–acceptor, pi‐sigma, pi‐sulfur	Ile50	2.57
				Ser49	2.26
				Met20	2.64
				Trp22	2.34
				Leu24	1.76
Cosmosiin	−7.6	Leu28, Met20	pi‐sigma, pi‐Alkyl	Leu24	1.98
				Ile50	2.56
				Ser49	2.28
cynaroside	−8.3	Leu28, Met20, Asn18	Unfavorable donor–donor, pi‐sigma, pi‐Alkyl	Asn18	1.99
				Ile50	2.03
				Ser49	2.36
				Trp22	2.86
Hesperetin	−8.2	Leu28, Met20	pi‐sigma, pi‐sulfur, pi‐Alkyl, Alkyl	Trp22	3.15
				Ile50	2.52
				Asp27	3.35
				Leu24	1.93
Luteolin	−7.5	Arg98, Asn28	Unfavorable donor–donor, pi‐cation, pi‐Alkyl	Arg98	2.6
				Asn28	2.86
				Glu110	3.05
				Thr123	2.73
Naringenin	−8.4	Leu28, Met20, Leu24	Unfavorable donor–donor, pi‐Sulfur, pi‐Sigma	Ser49	2.59
				Arg52	3.67
p‐Coumaric‐acid	−7.3	—	—	Met20	3.15
				Ser49	2.55
Protocatechuic‐aldehyde	−6.2	Met20	pi‐Alkyl	Met20	1.92
Quinic‐acid	−6.5	—	—	Ser49	2.04
				Trp22	2.21
				Met20	2.3
Salicylic‐acid	−6.6	Met20	pi‐Alkyl	Met20	2.3
				Trp22	2.89
Ascorbic acid	−6.3	—	—	Asn23	2.87
				Met20	1.84
				Trp22	2.6
**5fzn**					
Acacetin	−10.1	Ala366, Arg415, Ala556	Pi‐Alkyl, Alkyl	Gly464	3.62
				Val465	2.83
				Val512	2.21
				Val606	2.03
				Gly367	1.92
Apigenin	−9.9	Arg415, Ala556, Ala366	Unfavorable donor–donor, Pi‐Alkyl	Val512	2.26
				Val606	1.98
				Gly367	2.23
Caffeic‐acid	−7.3	Ala336	Unfavorable donor–donor, Pi‐Alkyl	Val512	2.15
				Val465	1.99
				Val418	2.37
				Lue557	2.08
				Ala556	3.43
Chlorogenic‐acid	−9.5	Thr560	Unfavorable donor–donor	Val606	1.97
				Thr560	2.45
				Lue557	2.19
				Gly509	3.65
				Val604	2.89
				Val465	2.16
				Gly417	3.51
Cosmosiin	−9.4	Ala363, Ile559, Cys513, Val561,	Unfavorable donor–donor, Pi‐Alkyl, Unfavorable acceptor–acceptor	Lue557	2.24
				Lue356	2.09
				Gly364	2.91
				Val463	2.57
				Val465	1.92
				Gly417	3.77
Cynaroside	−10.2	Ile559, Cys513, Lue557, Val561, Thr560	Unfavorable donor–donor, Pi‐Alkyl, Unfavorable acceptor–acceptor	Ile416	2.11
				Lue356	1.85
				Gly464	3.78
				Val604	1.97
				Val465	2.54
				Gly417	3.28
Hesperetin	−9.9	Ala366, Arg415, Ala556	Alkyl, Pi‐Alkyl, Unfavorable acceptor–acceptor	Gly367	1.89
				Val606	2.03
				Val465	3
				Val512	2.13
				Gly464	3.64
Luteolin	−10.3	Ala366	Pi‐Alkyl	Gly364	3.38
				Gly367	2.06
				Val606	2
				Val465	2.35
				Val512	1.96
				Gly464	3.31
Naringenin	−9.9	Ala366, Arg415, Ala556	Unfavorable donor–donor, Pi‐Alkyl	Gly464	3.68
				Gly367	1.85
				Val606	2.47
				Val512	2.04
p‐Coumaric‐acid	−6.7	Ala366, Gly367	Unfavorable donor–donor, Pi‐Sigma	Ile416	1.97
				Val463	2.97
				Gly509	3.77
Protocatechuic‐aldehyde	−6.7	Tyr525	Unfavorable donor–donor, Pi–Pi Staked	Ser508	2.8
				Arg415	2.84
				Arg483	2.78
				Ser555	1.93
				Gln530	1.94
Quinic‐acid	−7.1	—	—	Ala556	3.66
				Gly509	2.47
				Val604	2.52
				Ala366	3.79
				Leu365	2.63
Salicylic‐acid	−6.9	Tyr525, Arg483	Pi–Pi staked	Ser508	1.9
				Arg483	2.07
				Arg415	2.29
Ascorbic acid	−6.7	—	—	Ala510	2.5
				Val463	2.58
				Ile416	1.89

**FIGURE 6:**
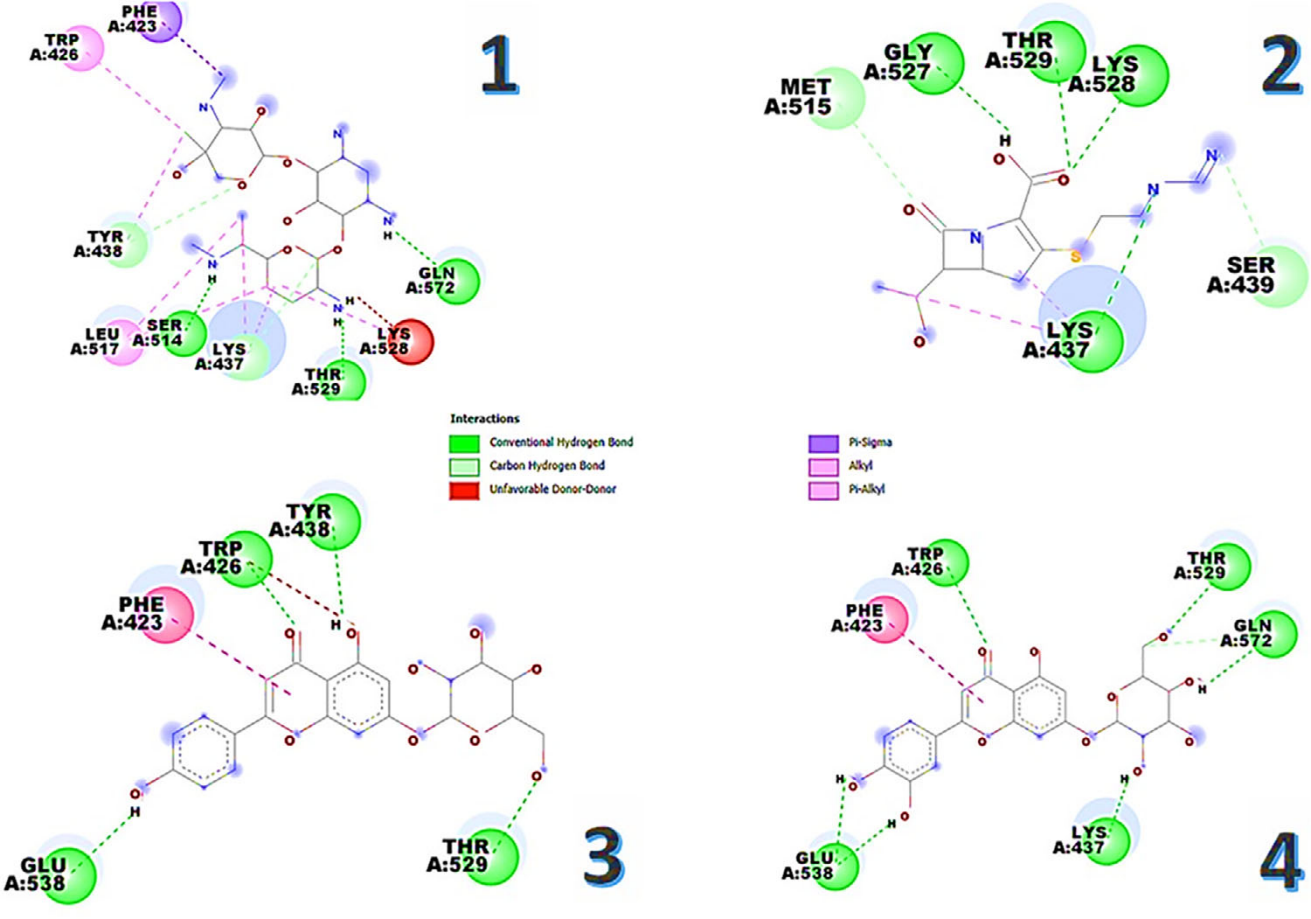
Representation of the favorable interaction of **6o9s**: (1): Gentamicin; (2): Imipenem; (3): Cosmosiin; (4): Cynaroside.

**FIGURE 7:**
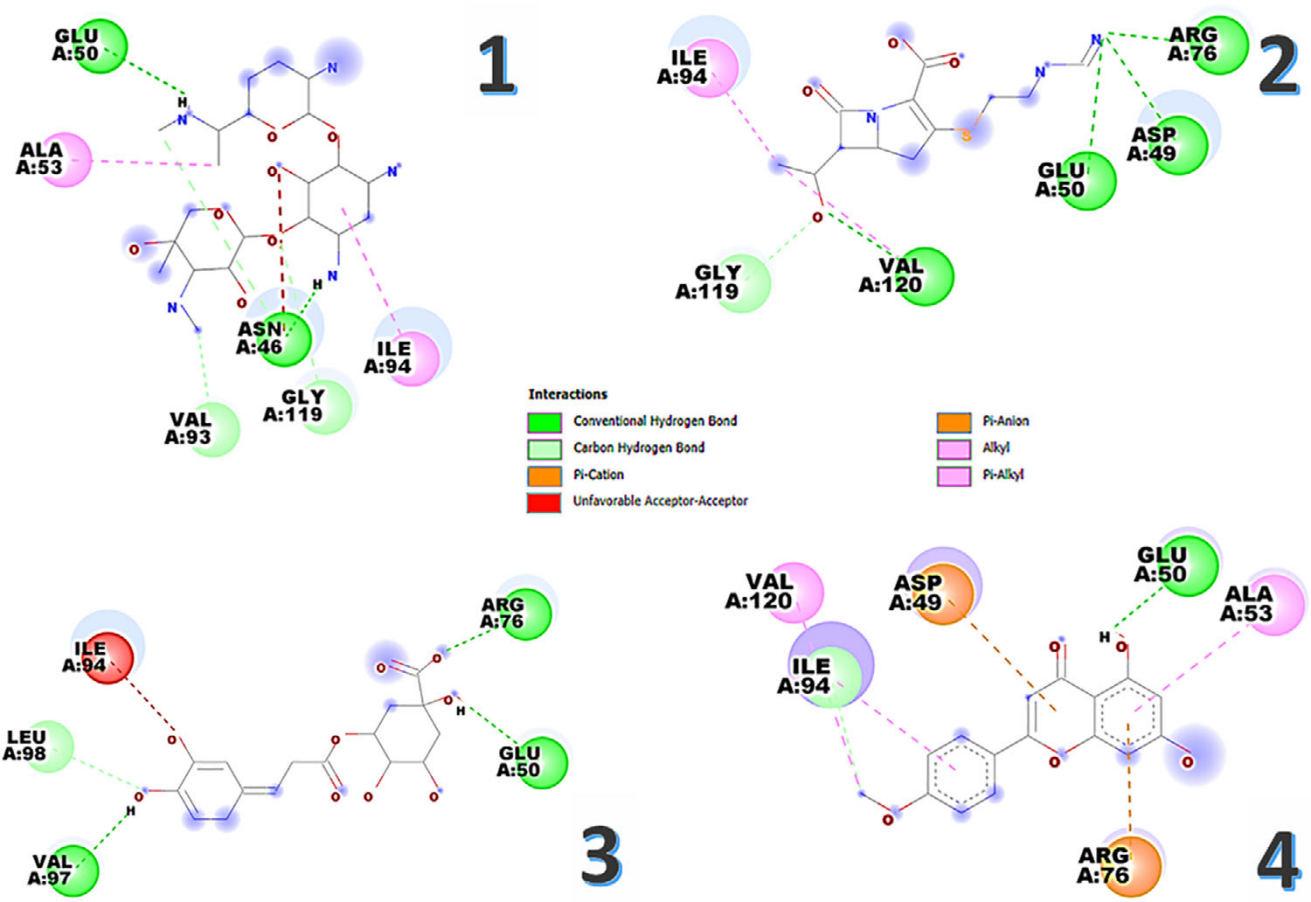
Representation of the favorable interaction of **7p2m**: (1): Gentamicin; (2): Imipenem; (3): Chlorogenic acid; (4): Acacetin.

**FIGURE 8:**
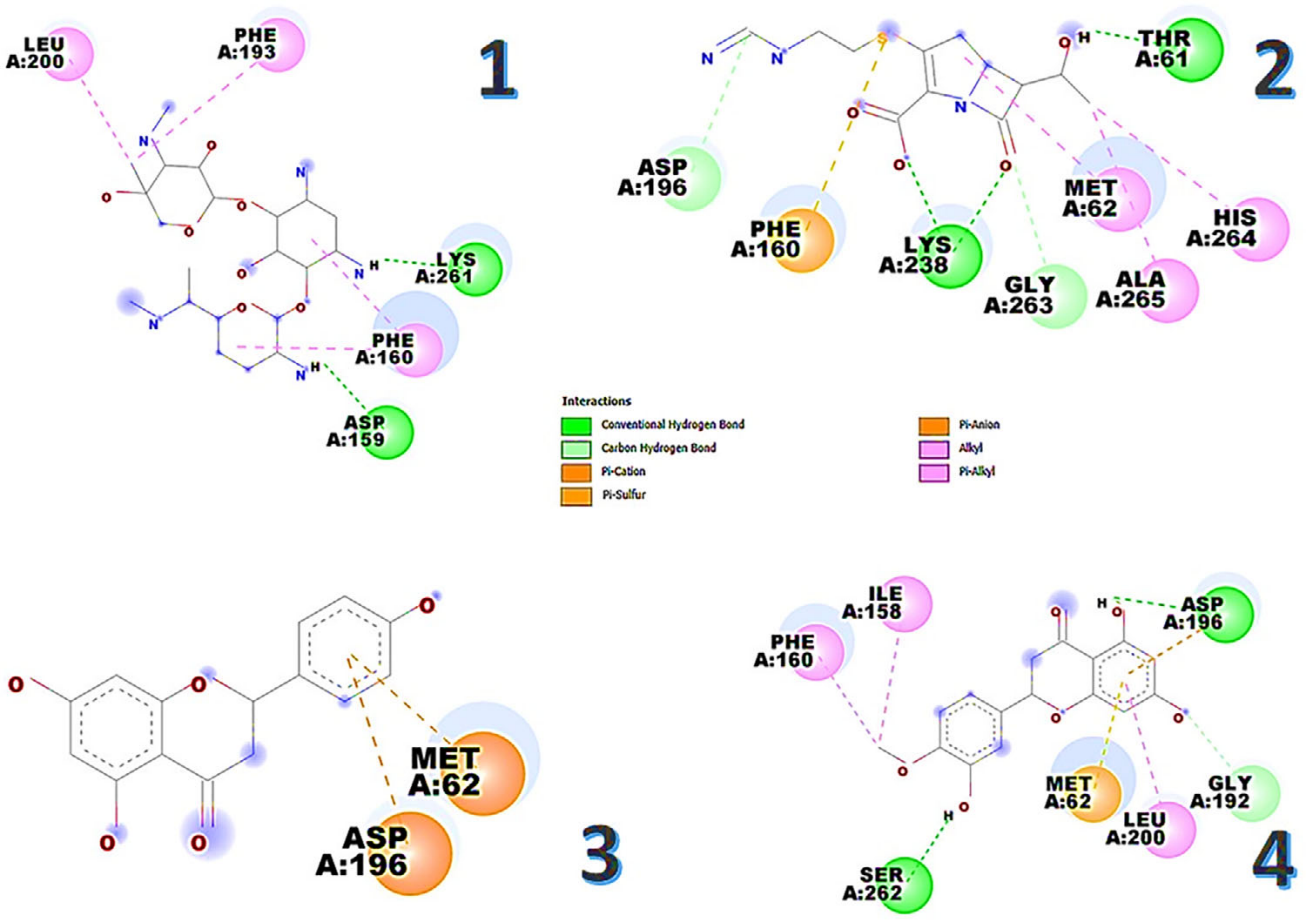
Representation of the favorable interaction of **6dui**: (1): Gentamicin; (2): Imipenem; (3): Naringenin; (4): Hesperetin.

**FIGURE 9:**
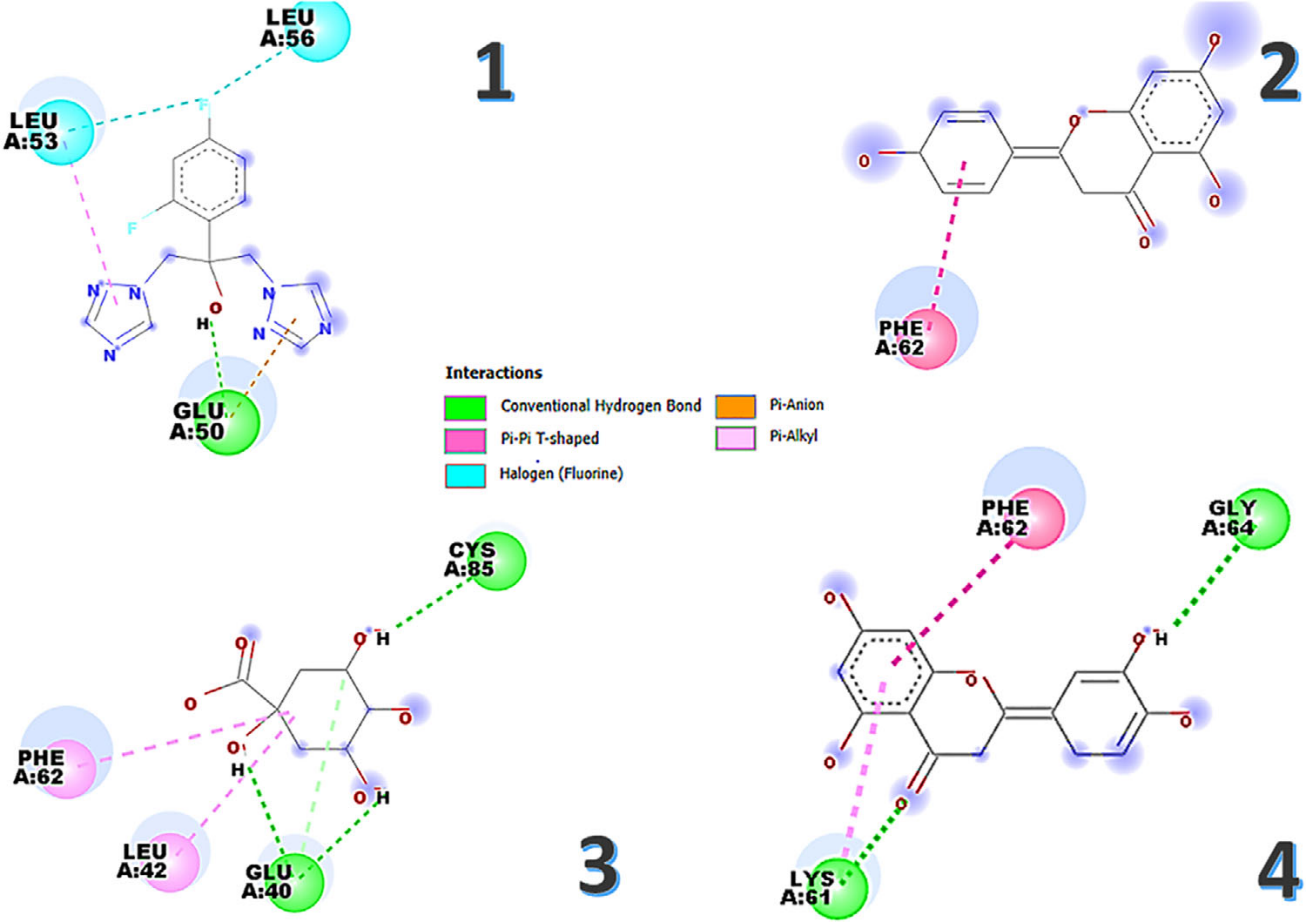
Representation of the favorable interaction of **7rh8**: (1): Fluconazole; (2): Apigenin; (3): Quinic acid; (4): Luteolin.

**FIGURE 10:**
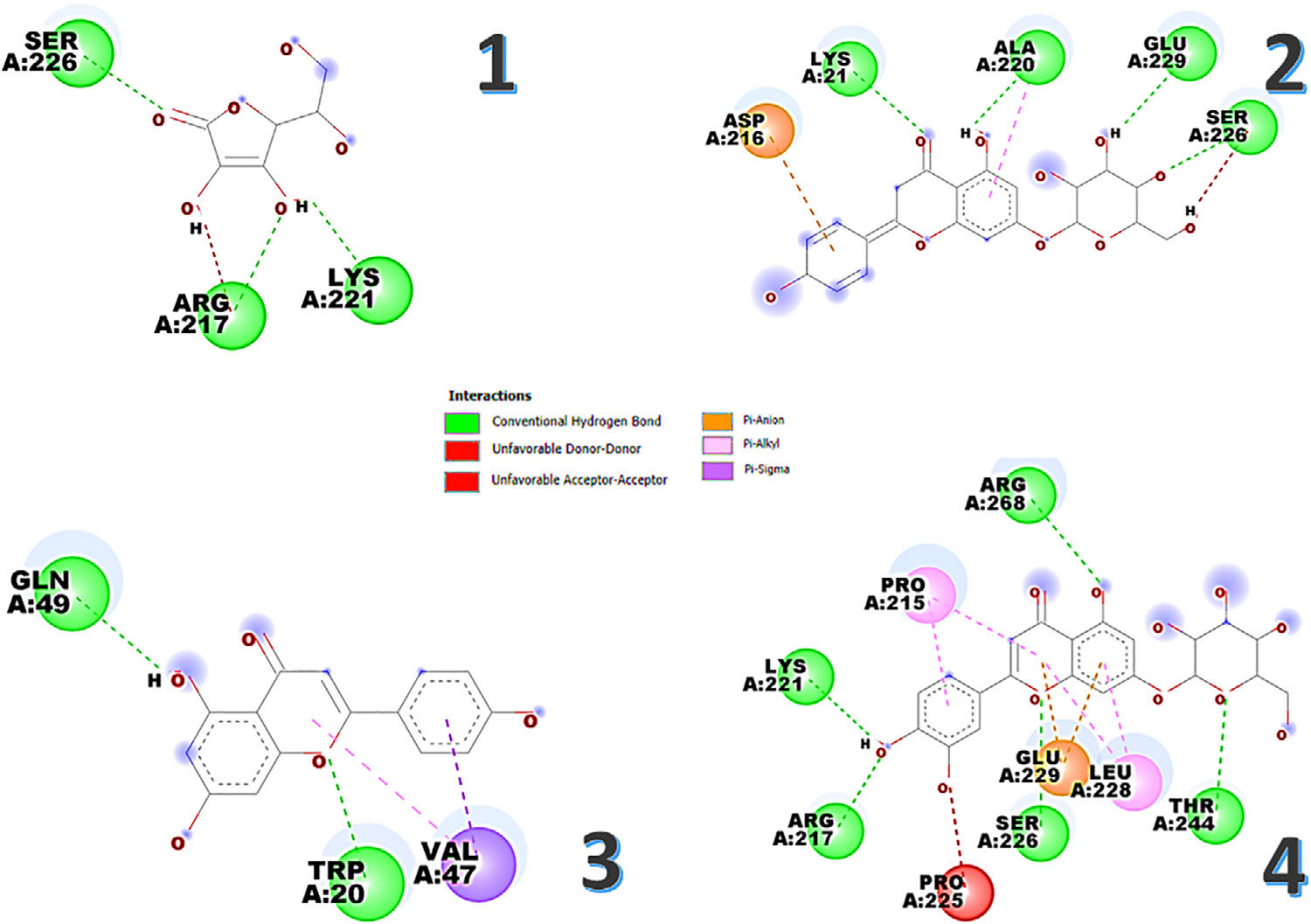
Representation of the favorable interaction of **8fh8**: (1): Ascorbic acid; (2): Cosmosiin; (3): Apigenin; (4): Cynaroside.

**FIGURE 11:**
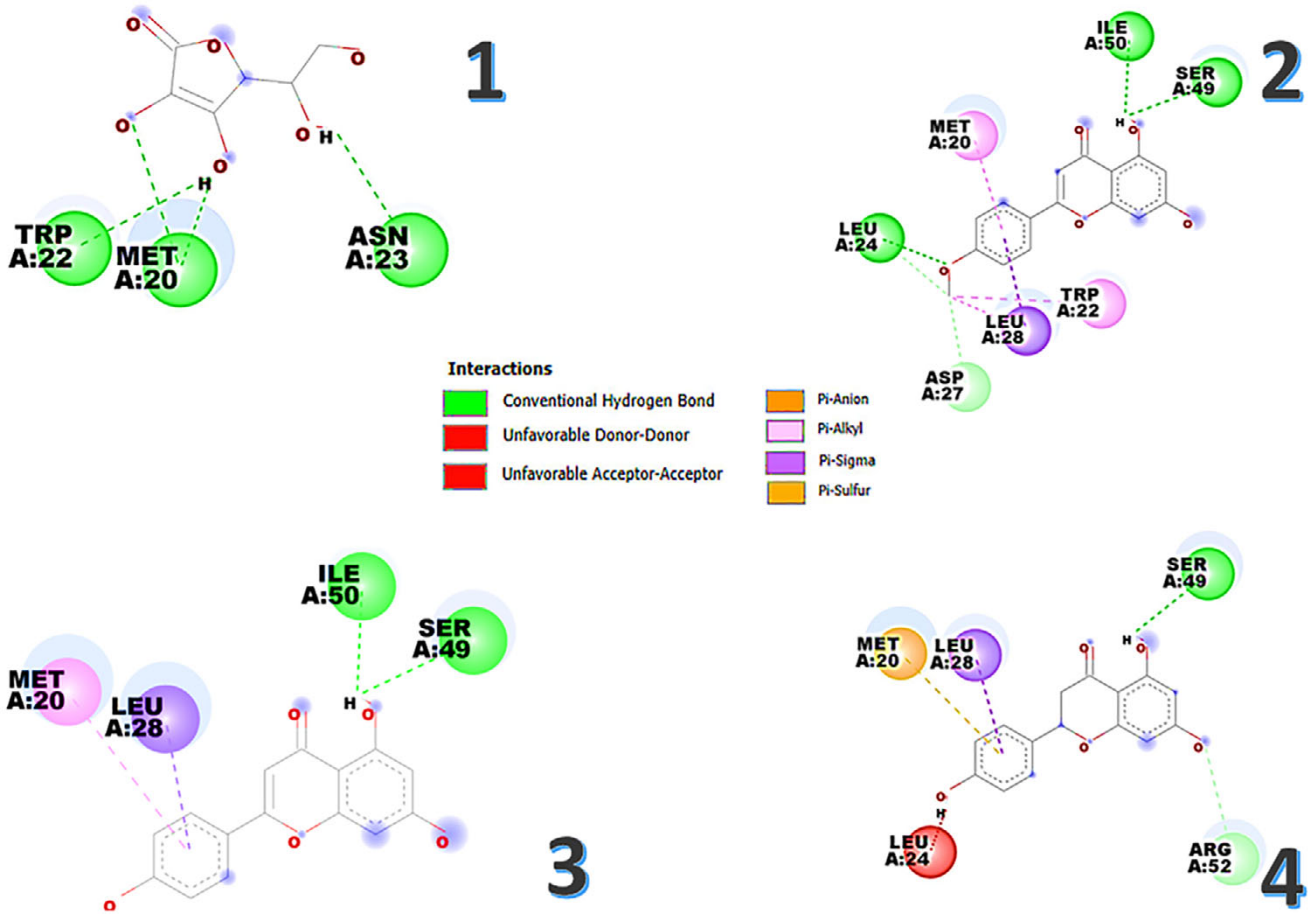
Representation of the favorable interaction of **5sss**: (1): Ascorbic acid; (2): Acacetin; (3): Apigenin; (4): Naringenin.

**FIGURE 12:**
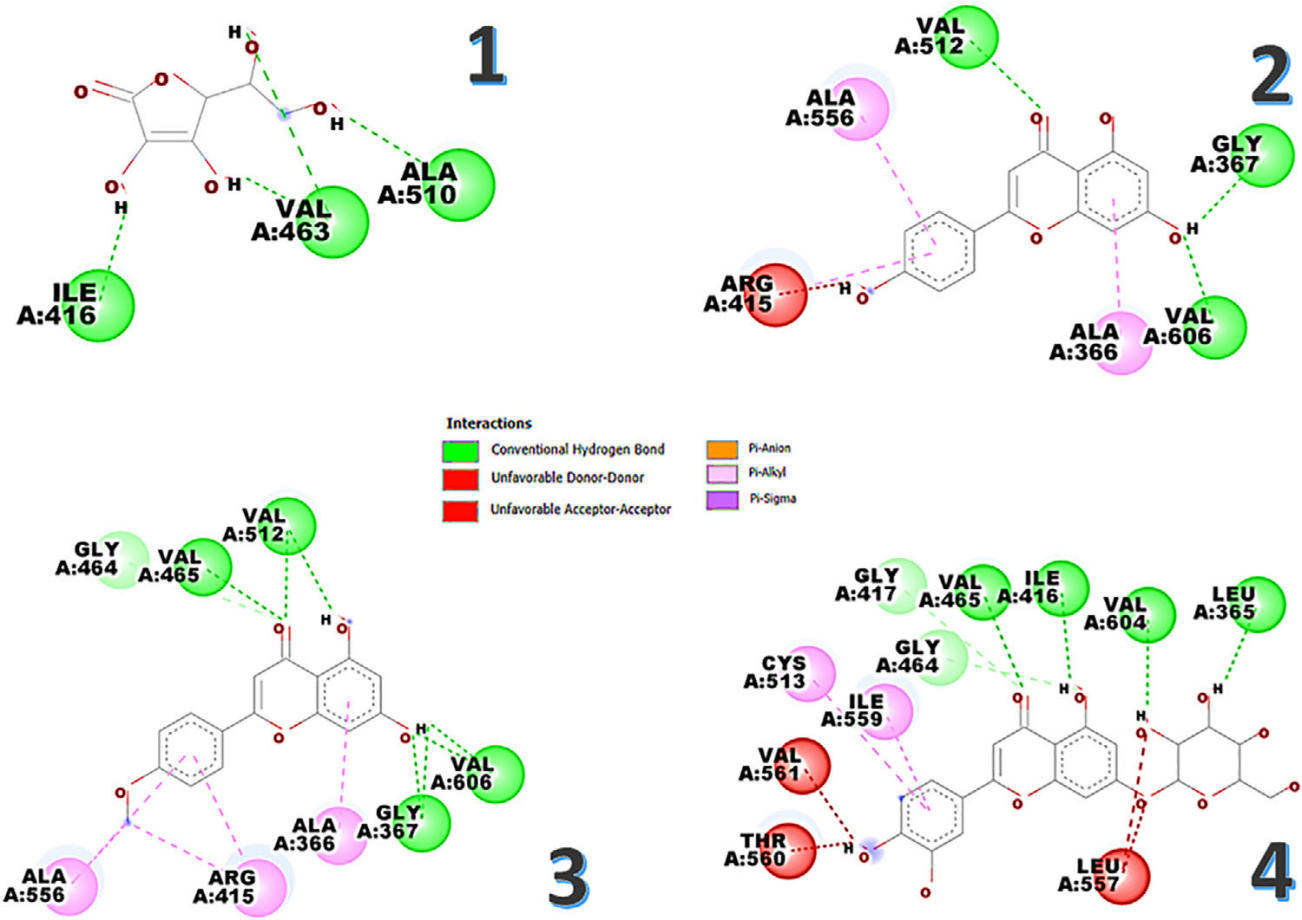
Representation of the favorable interaction of **5fzn**: (1): Ascorbic acid; (2): Apigenin; (3): Acacetin; (4): Cynaroside.

For the MecR1 target (6o9s), cosmosiin showed the maximum binding energy at −8.4 kcal/mol, characterized by many H‐bonds with main residues such as Tyr438, Thr529, and Glu538, alongside significant Pi–Pi T‐shaped hydrophobic interactions with Phe423. Cynaroside closely followed with −8.1 kcal/mol, exhibiting similarly strong interactions, including a notable H‐bond with Lys437. Apigenin also showed considerable affinity (−7.9 kcal/mol) through Pi–Pi stacking and short H‐bonds, particularly with Glu538. In contrast, salicylic acid displayed limited stability with a binding energy of −4.9 kcal/mol and unfavorable donor–donor interactions. Among standard drugs, gentamicin (−7.8 kcal/mol) showed strong binding, while Imipenem (−6.3 kcal/mol) exhibited moderate affinity. While against LpxC (6dui), cosmosiin again emerged as the strongest binder with −7.9 kcal/mol, forming H‐bonds with Lys261 and Tyr199, complemented by diverse hydrophobic interactions. Apigenin, chlorogenic acid, hesperetin, and naringenin also demonstrated high binding energies (−7.7 to −7.8 kcal/mol). Cynaroside, at −6.8 kcal/mol, formed stable H‐bonds with Lys238 and Ser262. Weaker binding was observed for acacetin (−5.2 kcal/mol) and caffeic acid (−5.6 kcal/mol). Reference drugs Gentamicin (−7.2 kcal/mol) and Imipenem (−7.0 kcal/mol) demonstrated good binding affinities. Against GyrB24 (PDB ID: 7p2m), chlorogenic acid exhibited the highest affinity at −8.6 kcal/mol, forming multiple hydrogen bonds with residues such as Arg76, Glu50, and Val97. Cynaroside also showed excellent binding (−8.4 kcal/mol) with five hydrogen bonds, indicating a stable complex. Acacetin (−7.4 kcal/mol) and apigenin (−7.3 kcal/mol) displayed good binding, whereas salicylic acid and protocatechuic aldehyde exhibited weaker interactions. Gentamicin (−6.8 kcal/mol) and Imipenem (−5.7 kcal/mol) served as reference points. For the Fur1p target (PDB ID: 7rh8), apigenin showed the strongest binding affinity at −7.6 kcal/mol, characterized by a stable π–π stacked interaction with Phe62. Cynaroside (−6.5 kcal/mol) and cosmosiin (−6.2 kcal/mol) also performed well, forming H‐bonds and hydrophobic contacts. Fluconazole, a reference drug, exhibited a distinct binding mode involving a halogen bond.

Meanwhile, against the oxidoreductase target AKR1B1(8fh8), cynaroside demonstrated the strongest binding affinity at −8.0 kcal/mol, forming five H‐bonds with residues such as Thr244, Ser226, and Lys221, indicating a highly stable multi‐point interaction. Apigenin (−7.8 kcal/mol) and cosmosiin (−7.5 kcal/mol) also showed strong binding with multiple H‐bonds. Weaker binding was observed for salicylic acid, quinic acid, and ascorbic acid. In addition, for the FOL (5sss), cynaroside again exhibited the strongest binding at −8.3 kcal/mol, forming five H‐bonds with residues like Asn18, Ile50, and Lys221. Apigenin, acacetin, and hesperetin followed closely at −8.2 kcal/mol, each forming multiple H‐bonds. Chlorogenic acid also bound strongly (−8.0 kcal/mol). Finally, against Nrf2 (5fzn), cynaroside showed the strongest binding affinity at −10.2 kcal/mol, forming six H‐bonds with key residues such as Ile416, Lue356, and Val604. Luteolin followed closely with −10.3 kcal/mol, creating several short H‐bonds. Acacetin and apigenin also demonstrated strong affinities (−10.1 and −9.9 kcal/mol, respectively). Overall, the flavonoids with extended conjugation and hydroxylation (apigenin, luteolin, naringenin, hesperetin) achieve strong binding through combined aromatic stacking and hydrogen bonding, simple phenolic acids (caffeic, coumaric, protocatechuic, salicylic) show weaker binding due to limited interaction sites; antibiotics like gentamicin exploit their polyhydroxylated frameworks for strong stabilization, whereas imipenem's β‐lactam scaffold yields moderate but diverse interactions. However, cosmosiin and cynaroside demonstrated the highest potency. Their enhanced binding affinity results from the 7‐O‐glucoside moiety, which substantially increases the hydrogen‐bonding network within protein active sites. Additionally, the catechol B‐ring in cynaroside confers an electronic advantage for oxidative stress‐related targets (Nrf2/AKR1B1), promoting stronger electrostatic interactions and greater thermodynamic stability compared to the corresponding aglycones and simpler phenolic derivatives. These results are supported by previous studies that have found that Docking results for these two compounds are always higher than those for other compounds [65, 66, 67, 68]. However, the limitations of in silico analyses should be acknowledged, as these methods often assume proteins are rigid, neglect solvent effects, and omit molecular dynamics simulations. Such simplifications may reduce the accuracy of docking predictions, underscoring the importance of cautious interpretation and complementary experimental validation [69, 70].

### 3.5 Correlation Between Phenolics, Antioxidant and Antimicrobial Activities

The results in Table 8 show the correlation between the phenolic compounds found in the extract, antioxidant activities, and antimicrobial parameters, each supported by statistically significant *p*‐values (*p* < 0.001).

**TABLE 8:** Pearson correlation coefficient (*r*) between different assays of extracts.

Variables	Protocatechuic aldehyde	Cynaroside	Cosmosiin	TPC	DPPH	FRAP	ABTS	MIC_50_‐*SA*	MIC_90_‐*SA*	MIC_50_‐*PA*	MIC_50_‐*EC*	MIC_90_‐*EC*	MBC‐EC	MIC_50_‐*CA*	MIC_90_‐*CA*	MBC‐*CA*
Protocatechuic aldehyde	**1**															
Cynaroside	**0.9044**	**1**														
Cosmosiin	**0.8894**	**0.9980**	**1**													
TPC	−0.5741	−0.7080	−0.7490	**1**												
TFC	0.1918	−0.2157	−0.2632	0.5429												
DPPH	−**0.6606**	−0.2790	−0.2443	−0.0066	**1**											
FRAP	−0.1473	−0.2170	−0.2729	**0.8393**	−0.1274	**1**										
ABTS	−**0.6241**	−0.2593	−0.2133	−0.1748	**0.9715**	−0.3534	**1**									
MIC_50_‐*SA*	−**0.6469**	−0.2905	−0.2795	0.2889	**0.9166**	0.2735	**0.7958**	**1**								
MIC_90_‐*SA*	−**0.6131**	−0.2468	−0.2354	0.2550	**0.9173**	0.2613	**0.7970**	**0.9990**	**1**							
MBC‐*SA*	−**0.6088**	−0.2413	−0.2299	0.2508	**0.9172**	0.2597	**0.7971**	**0.9987**	**1.0000**							
MIC_50_‐*PA*	−0.2420	−**0.5167**	−**0.4954**	−0.0164	−0.3068	−0.4806	−0.1363	−0.5436	−0.5724	**1**						
MIC_90_‐*PA*	−0.2606	−**0.5373**	−**0.5169**	0.0095	−0.3025	−0.4602	−0.1362	−0.5324	−0.5621	**0.9997**						
MBC‐*PA*	−0.2630	−**0.5399**	−**0.5196**	0.0128	−0.3019	−0.4575	−0.1362	−0.5309	−0.5608	**0.9996**						
MIC_50_‐*EC*	−0.3681	−0.3175	−0.3615	**0.8353**	0.1953	**0.9477**	−0.0365	0.5639	0.5515	−0.5623	**1**					
MIC_90_‐*EC*	−0.3561	−0.3038	−0.3481	**0.8286**	0.1919	**0.9489**	−0.0407	0.5621	0.5502	−0.5727	**0.9999**	**1**				
MBC‐*EC*	−0.3546	−0.3021	−0.3464	**0.8277**	0.1915	**0.9491**	−0.0412	0.5619	0.5501	−0.5740	**0.9999**	**1.0000**	**1**			
MIC_50_‐*CA*	0.5113	**0.5429**	**0.5860**	−**0.9546**	−0.1294	−**0.9251**	0.0795	−0.4719	−0.4478	0.3096	−**0.9603**	−**0.9567**	−**0.9562**	**1**		
MIC_90_‐*CA*	0.5361	**0.5481**	**0.5893**	−**0.9459**	−0.1765	−**0.9137**	0.0327	−0.5135	−0.4898	0.3268	−**0.9641**	−**0.9603**	−**0.9598**	**0.9988**	**1**	
MBC‐*CA*	0.5391	**0.5487**	**0.5897**	−**0.9446**	−0.1824	−**0.9121**	0.0269	−0.5186	−0.4949	0.3289	−**0.9644**	−**0.9606**	−**0.9601**	**0.9985**	**1.0000**	**1**

Bold values are different from 0 at a significance level alpha = 0.05.

The observed negative correlation between phenolic contents and their biochemical activities indicates that the compound's concentration is inversely proportional to the measured activity (EC_50_). Thereby, significant negative correlations were found between protocatechuic aldehyde and the following measures: DPPH (*r* = ‐0.6606, *p* = 0.03394), ABTS (*r* = −0.6241, *p* = 0.0376), MIC_50%_‐*SA* (*r* = −0.6469, *p* = 0.0353), MIC_90%‐_ *SA* (*r* = −0.6131, *p* = 0.0387), and MBC‐*SA* (*r* = −0.608, *p* = 0.0391). These findings suggest that protocatechuic aldehyde contributes to the observed antibacterial and antioxidant activities, as demonstrated in recent studies [71, 72, 73]. Similarly, there are moderately significant negative correlations for cynaroside and cosmosiin with MIC_50%_‐*PA* (*r* = −0.5167, *r* = −0.4954, *p* = 0.04833, 0.0505), MIC_90%_‐*PA* (*r* = −0.5373, *r* = −0.5169, *p* = 0.04627, 0.0483) and MBC‐*PA* (*r* = −0.5399, *r* = −0.5196, *p* = 0.04601, 0.0480), respectively. These results demonstrate that these two compounds are responsible for over 50% of the observed anti‐*PA* activity. These results are supported by previous studies on the antibacterial effectiveness of cynaroside and cosmosiin [74, 75]. Moreover, the positive correlation of these two compounds with MIC_50%_‐*CA* (*r* = 0.5429, *r* = 0.5860, *p* = 0.0457, 0.0414), MIC_90%_‐*CA* (*r* = 0.5481, *r* = 0.5893, *p* = 0.0452, 0.0411) and MBC‐*CA* (*r* = 0.5487, *r* = 0.5897, *p* = 0.0451, 0.0410) explain that the varying levels of cynaroside and cosmosiin in the extracts are linked to more than 54% of their antibacterial ineffectiveness.

On the other hand, the total phenolic content (TPC) shows a strong positive correlation with FRAP antioxidant activity (*r* = 0.8393, *p* = 0.0472), confirming that higher phenolic levels enhance antioxidant capacity. This result is in agreement with those found by Kiss et al. [76] and Heckmann et al. [77], that they found a strong positive correlation between FRAP and TPC (*r* = 0.913, *p* = 0.001) and (*r* = 0.8572, *p* < 0.0001), respectively.

The ABTS and DPPH assays are also strongly correlated (*r* = 0.9715, *p* = 0.0285), demonstrating consistency between these two methods. Several studies support this finding. Goudjil et al. [78] showed a good correlation between ABTS and DPPH, with *r* = 0.7201 and *p* < 0.001, in their research on the butanoic extract of *Crataegus monogyna Jacq*. In other research, they also found a very strong correlation between DPPH and ABTS in 12 plant species, with *r* = 0.916 and *p* < 0.001 [79]. Additionally, another study reported a strong correlation between ABTS and DPPH, with *r* = 0.961 and *p* = 0.002 [80].

In terms of antimicrobial activity, MIC_50%_‐*SA* and MIC_90%_‐*SA* are nearly perfectly correlated (*r* = 0.9990, *p* = 0.0010), and MBC‐*SA* is perfectly correlated with MIC_90%_‐*SA* (*r* = 1.0000, *p* < 0.0001), showing that inhibitory and bactericidal concentrations are closely aligned. For *P. aeruginosa*, MIC_90%_‐*PA* and MBC‐*PA* are also perfectly correlated (*r* = 1.0000, *p* < 0.0001), with MIC_50%_‐PA showing a strong correlation as well (*r* = 0.9997, *p* = 0.0003). In the case of *E. coli*, MIC_90%_‐*EC* and MBC‐*EC* are perfectly correlated (*r* = 1.0000, *p* < 0.0001), and TPC shows a positive correlation with these values (*r* ≈ 0.8286‐0.8353, *p* ≈ 0.045‐0.049. For *C. albicans*, TPC shows strong negative correlations with MIC_50%_‐*CA* (*r* = −0.9546, *p* = 0.0457), MIC_90%_‐*CA* (*r* = −0.9459, *p* = 0.0554), and MBC‐*CA* (r = −0.9446, *p* = 0.0451). Moreover, MIC_50%_‐*SA*, MIC_90%_‐*SA*, and MBC‐*SA* showed strong significant positive correlations with ABTS (*r* = 0.795, *r* = 0.7970 and *r* = 0.7971, *p* = 0.0492) and even more so with DPPH (*r* = 0.9166, *r* = 0.9173 and *r* = 0.9172, *p* = 0.0483). While FRAP showed as strong positive correlation with MIC_50%_‐*EC* (*r* = 0.9477, *p* = 0.0452), MIC_90%_‐*EC* (*r* = 0.9489, *p* = 0.0451) and MBC‐*EC* (*r* = 0.9491, *p* = 0.0451), and showed as strong negative correlation with MIC_50%_‐*CA* (*r* = −0.9251, *p* = 0.0475), MIC_90%_‐*CA* (*r* = −0.9137, *p* = 0.0486) and MBC‐*CA* (*r* = −0.9121, *p* = 0.0488). This correlation suggests that the compounds contributing to the antioxidant activity of these plants also serve as effective inhibitors against *S. aureus* and *E. coli*. Several studies confirm that some plant extracts function as both antioxidants (FRAP, ABTS, DPPH) and antimicrobials against *E. coli* and *S. aureus* [81, 82]. Moreover, in a study of the aqueous extract of *Salvia blancoana subsp. Mesatlantica*, the FRAP and MIC/MBC‐*CA* are inversely correlated [83].

Additionally, in a recent study, the MIC‐*EC* and MIC‐*SA* showed a good positive correlation between them (*r* = 0.75, *p*>0.05) and a good negative correlation with DPPH (*r* = −0.76, *p* = 0.13, *r* = −0.57, *p* = 0.32) and TPC (*r* = −0.77, *p* = 0.13, *r* = −0.73, *p* = 0.16), respectively [84]. Therefore, Luisa Pozzo et al. found a negative Pearson's correlation between MIC, MBC‐*SA* and MIC, MBC‐*EC* (*r* = −0.617, *p *< 0.05) and a strong positive correlation between MIC‐*EC* and TPC (*r* = 0.812, *p* < 0.05) and a good correlation between FRAP and MIC, MBC‐*EC* and DPPH with MIC, MBC‐*EC* (*r* = 0.641, *r* = 0.730, *p* < 0.05) [85].

To better understand the similarities and differences among the studied plants, a collection analysis was conducted using principal component analysis (PCA) based on the data matrix of four extracts and eight methods. The PCA biplot (F1 = 50.12%, F2 = 26.27%, total 76.39%) illustrates the relationships between phytochemical composition, antioxidant capacity, and antimicrobial activity of the different extracts (AH_Dc_, Md_Dc_, AH_Aqu_, and Md_Aqu_), which are highlighted by circles in Figure 13.

**FIGURE 13:**
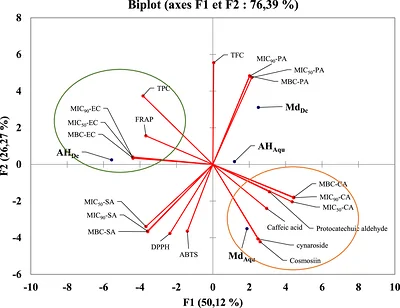
The PCA biplot showing the correlation between TPC, TFC, and the biological activities of the extracts.

Initially, the green circle groups AH_Dc_ with *E. coli* inhibition variables (MIC_50%_‐*EC*, MIC_90%_‐*EC*, MBC‐*EC*) and FRAP/TPC, showing its association with phenolic content and antimicrobial activity against *E. coli*. While the blue circle contains Md_Dc_, closely related to TFC and antimicrobial activity against *P. aeruginosa* (MIC_50%_‐*PA*, MIC_90%_‐*PA*, MBC‐*PA*), which confirms that this extract is rich in flavonoids and selectively effective against *P. aeruginosa*. Moreover, the orange circle associates Md_Aqu_ with phenolic compounds (caffeic acid, protocatechuic aldehyde, cynaroside, cosmosiin) and *C. albicans* inhibition variables (MIC_50%_‐*CA*, MIC_90%_‐*CA*, MBC‐*CA*), which designates the strong antioxidant profile and antifungal properties. AH_Aqu_ is positioned near the center and is weakly linked to most variables.

UHPLC‐MS quantification and molecular docking analyses revealed an apparent relationship between compound abundance and predicted binding affinity. Acacetin emerged as the most abundant identified compound (485 mg/g extract) and exhibited favorable predicted binding affinities, ranging from −7.4 to −10.1 kcal/mol. The combination of its high abundance and favorable docking scores suggests that acacetin may be an important contributor to the biological activity of the extract. Cosmosiin also showed moderate‐to‐high abundance (75–213 mg/g) and favorable predicted binding affinities (−7.2 to −9.4 kcal/mol), suggesting its potential contribution to the antimicrobial and antioxidant activities of the extract. In contrast, quinic acid, despite its relatively high abundance (262 mg/g), exhibited weaker predicted binding affinities (−5.5 to −7.1 kcal/mol). These results suggest that quinic acid may play a less prominent role as a direct target binder, although its potential contribution to the overall biological activity of the extract cannot be excluded. Moreover, the presence of significant negative correlations between cynaroside and cosmosiin levels and their antimicrobial activities against *P. aeruginosa* (*r* > −0.54, *p* < 0.002), on the one hand, and the presence of strong binding affinities between these two compounds and the selected microbial targets measured by molecular docking, on the other, suggest the existence of a concordance between the in vitro and in silico results. Nevertheless, this association remains indicative and should be experimentally validated through further in vitro and in vivo studies investigating the direct antimicrobial effects and mechanisms of action of cynaroside and cosmosiin.

In other side, Table 9 highlighted the strong, statistically significant correlations between the protein targets in the docking study, for example, 6o9s shows very strong correlations with 7p2m (*r* = 0.8815, *p* < 0.0001), 7rh8 (*r* = 0.9231, *p* < 0.0001), 8fh8 (*r* = 0.9289, *p* < 0.0001) and 5fzn (*r* = 0.8968, *p* < 0.0001), indicating these variables are highly associated. Similarly, 7p2m is strongly correlated with 8fh8 (*r* = 0.8945, *p* < 0.0001), 5sss (*r* = 0.8606, *p* = 0.0002), and 5fzn (*r* = 0.8563, *p* = 0.0002). 7rh8 also shows strong links with 8fh8 (*r* = 0.9033, *p* < 0.0001) and 5fzn (*r* = 0.8535, *p* = 0.0002). Furthermore, 8fh8 is significantly correlated with 5sss (*r* = 0.8462, *p* = 0.0003) and 5fzn (*r* = 0.8755, *p* < 0.0001), while 5sss and 5fzn themselves share a strong correlation (*r* = 0.8274, *p* = 0.0005). These high *R*‐values combined with low *p*‐values (mostly < 0.0001) confirm that the relationships are not due to chance. Globally, the variables 6o9s, 7p2m, 7rh8, 8fh8, 5sss and 5fzn form a tightly connected group with strong and statistically significant correlations, whereas 6dui appears less integrated,

**TABLE 9:** Pearson correlation coefficient (*r*) between different targets using in the docking study.

Variables	6o9s	6dui	7p2m	7rh8	8fh8	5sss	5fzn
6o9s	**1**						
6dui	**0.6819**	**1**					
7p2m	**0.8815**	0.5313	**1**				
7rh8	**0.9231**	**0.6578**	**0.7810**	**1**			
8fh8	**0.9289**	0.4841	**0.8945**	**0.9033**	**1**		
5sss	**0.7792**	0.4605	**0.8606**	**0.7233**	**0.8462**	**1**	
5fzn	**0.8968**	**0.6037**	**0.8563**	**0.8535**	**0.8755**	**0.8274**	**1**

Bold values are different from 0 at a significance level alpha = 0.05.

## 4 Conclusions

This investigation, employing in vitro bioassays alongside complementary in silico studies, provides a scientific approach for the traditional use of *Atriplex halimus L*. and *Marrubium deserti* De Noé in Algerian folk medicine. Both plant's dichloromethane extracts demonstrated significant antioxidant activity and notable antimicrobial efficacy, particularly against *C. albicans*. The in silico analysis further supported these findings by elucidating plausible molecular mechanisms of action and identifying specific compounds with favorable interactions with relevant biological targets. These findings support the therapeutic potential of these species and underscore the isolation, structural characterization, and eventual standardization of active principles for future pharmacological and clinical development.

## Author Contributions

**Nacira Siad**, **Chahrazed Hamia**, **Amar Djeridane** and **Mohamed Benalia**: experimental acquisition, writing – original draft, investigation, formal analysis, methodology, and data curation. **Reguia Mahfoudi**, **Hicham Bouakkaz**, **Mustafa Abdullah Yilmaz**, **Oguz Cakir**, **Abbas Tarhan**: LC‐MS‐MS analysis. **Mohamed Yousfi**: resources and funding acquisition.

## Funding

This research was financially supported by a grant from the Laboratory of Fundamental Sciences, Amar Telidji University. PB 37G, 03000, Laghouat, Algeria.

## Conflicts of Interest

The authors declare no conflicts of interest.

## Data Availability

The data that support the findings of this study are available from the corresponding author upon reasonable request.
